# Supplementary material for: Evaluating the clinical relevance and reliability of outer retinal band length on optical coherence tomography in retinal disease: a cross-sectional study
Source: BMJ Open. 2023 Dec 12;13(12):e077874. doi: 10.1136/bmjopen-2023-077874 (PMC10729289; doi:10.1136/bmjopen-2023-077874)
Supplement: Supplementary data [file bmjopen-2023-077874supp001.pdf]

Supplementary Material

Methods – OCT B-scan grading instructions

OCT B-scans were graded using the following instructions: '*Not counting the area directly under the optic nerve (if your image contains an optic nerve), what percentage of the horizontal length of the scan shows three complete and continuous hyperreflective lines in the retinal layers between the myoid zone and choriocapillaris? Ignore padding between around the OCT B-scan, shadowing and areas under the optic nerve.*' All images were reviewed in full-screen mode at 100% magnification, maximal brightness and standard contrast settings (Asus Vivobook S15).

Table S1: Diagnosis classification

Diagnosis category	Criteria for inclusion
Glaucoma disease	Glaucoma diagnosis recorded in clinical record
Glaucoma suspect	• Glaucoma risk requiring further work-up due to historical risk factors and/or detection of structural and/or functional signs suggestive of glaucoma.<br>• Narrow angles requiring further monitoring
Optic nerve disease	Non-glaucomatous optic nerve pathology
Macular disease	• AMD<br>• Pachychoroid spectrum disease<br>• Other macular pathology e.g. retinal dystrophy, epiretinal membranes<br>• Non-specific macular pathology e.g. isolated pigmented disruption or drusen
Retinal disease	• Diabetic retinopathy<br>• Myopia-related complications<br>• Extra-macular retinal pathology unrelated to myopia or diabetes
Other	• Corneal degenerations<br>• Visual symptoms or headaches unexplained by clinical findings<br>• Pigmented lesion<br>• Anterior eye disease including dry eye, conjunctival lesions or other pathology unrelated to the cornea<br>• Vitreous anomalies
Multiple diagnoses	Diagnosed with multiple conditions in different categories
No ocular pathology	No ocular pathology noted in clinician report including druplets, cataracts, refractive errors and physiological cupping or nerve crowding.
Outer retinal disease	Diagnosis of macular and/or retinal disease

Cheung R, *et al. BMJ Open* 2023; 13:e077874. doi: 10.1136/bmjopen-2023-077874

Table S2: Patient diagnoses

**Diagnostic category**	(**%**, n/N)	Foveal HORB length (mean, SD)	Non-foveal HORB length (mean, SD)
Glaucoma suspect	23.8 (143/600)	27.9 (27.4)	18.2 (24.8)
Macular disease	16.5 (99/600)	21.9 (26.4)	16.0 (21.8)
Retinal disease	12.0 (72/600)	25.5 (29.4)	20.3 (26.7)
Multiple diagnoses	10.8 (65/600)	21.8 (24.6)	16.8 (20.3)
Other diagnoses	9.3 (56/600)	31.1 (29.7)	23.5 (28.0)
Optic nerve disease	4.5 (27/600)	30.2 (25.3)	22.0 (21.4)
Glaucoma disease	3.5 (21/600)	14.9 (19.1)	9.4 (19.5)
No ocular pathology	19.5 (117/600)	28.9 (28.2)	22.2 (26.9)

Figure S1: pie chart of OCT scan patterns from which B-scans were extracted. *Other scan types include B-scans from Spectralis 20x20 scan patterns and single line B-scans.

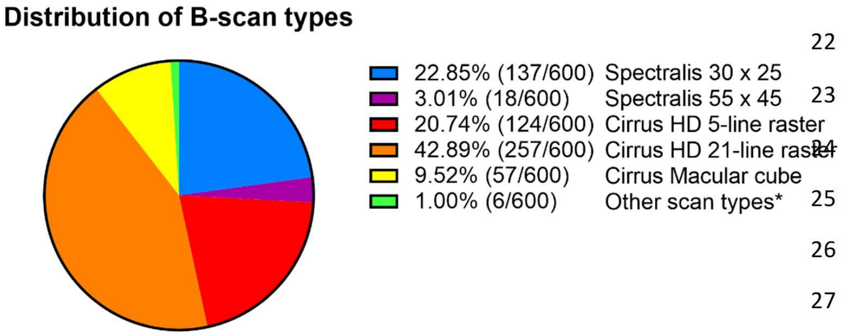

Table S3: Association between patient/scan characteristics and HORB length for patients without ocular pathology

**Univariable analysis**	**Foveal B-scans**		**Non-foveal B-scans**	
	p	*β*	*p*	*β*
Age (years)	**0.03**	-0.20	**<0.0001**	-0.22
Sex (reference: female)	0.80	-0.02	0.41	-0.08
VA (logMAR)	0.09	-0.16	0.06	-0.08
BVS (Dioptres)	0.89	0.01	**0.01**	0.12
B-scan length (mm)	0.69	-0.04	0.28	-0.04
Eccentricity (mm from fovea)	-	-	0.93	0.01
Device type[‡] (reference: Spectralis OCT)	0.84	-0.02	0.13	0.14
**Diagnosis** (reference: absence)				
Glaucoma disease	-	-	-	-
Glaucoma suspect	-	-	-	-
Macular disease	-	-	-	-
Retinal disease	-	-	-	-

Cheung R, *et al. BMJ Open* 2023; 13:e077874. doi: 10.1136/bmjopen-2023-077874

Optic nerve disease	-	-	-	-
Outer retinal disease	-	-	-	-
**Multiple linear regression analysis**				
Age (years)	**0.03**	-0.16	**0.05**	-0.20
VA (logMAR)	0.09	-0.16	0.38	-0.09
BVS (Dioptres)	-	-	**0.04**	0.21
B-scan length (mm*)*	-	-	-	-
Eccentricity (mm from fovea)	-	-	-	-
Device type (reference: Spectralis OCT)	-	-	0.18	0.13
**Diagnosis** (reference: absence)	-	-	-	-
Glaucoma disease				
Macular disease	-	-	-	-
Outer retinal disease	-	-	-	-

Cheung R, *et al. BMJ Open* 2023; 13:e077874. doi: 10.1136/bmjopen-2023-077874
