# Supplementary material for: Evaluating the clinical relevance and reliability of outer retinal band length on optical coherence tomography in retinal disease: a cross-sectional study
Source: BMJ Open. 2023 Dec 12;13(12):e077874. doi: 10.1136/bmjopen-2023-077874 (PMC10729289; doi:10.1136/bmjopen-2023-077874)
Supplement: Author's manuscript [file bmjopen-2023-077874.draft_revisions.pdf]

BMJ Open

BMJ Open is committed to open peer review. As part of this commitment we make the peer review history of every article we publish publicly available.

When an article is published we post the peer reviewers' comments and the authors' responses online. We also post the versions of the paper that were used during peer review. These are the versions that the peer review comments apply to.

The versions of the paper that follow are the versions that were submitted during the peer review process. They are not the versions of record or the final published versions. They should not be cited or distributed as the published version of this manuscript.

BMJ Open is an open access journal and the full, final, typeset and author-corrected version of record of the manuscript is available on our site with no access controls, subscription charges or pay-per-view fees (http://bmjopen.bmj.com).

If you have any questions on BMJ Open's open peer review process please email info.bmjopen@bmj.com

BMJ Open

The reliability and clinical relevance of outer retinal band length on OCT in retinal disease

Journal:	*BMJ Open*
Manuscript ID	bmjopen-2023-077874
Article Type:	Original research
Date Submitted by the Author:	18-Jul-2023

Complete List of Authors:	Cheung, Rene; University of New South Wales Faculty of Science, School of Optometry and Vision Science; UNSW Centre for Eye Health<br>Ly, Angelica; University of New South Wales Faculty of Science, School of Optometry and Vision Science; UNSW Centre for Eye Health<br>Wang, Henrietta; UNSW Centre for Eye Health; University of New South Wales, Optometry and Vision Science<br>Kalloniatis, Michael; University of New South Wales, School of Optometry and Vision Science; Deakin University<br>Nivison-Smith, Lisa; University of New South Wales Faculty of Science, School of Optometry and Vision Science

Keywords:	Medical retina < OPHTHALMOLOGY, Diagnostic Imaging, Primary Health Care, Retrospective Studies

SCHOLARONE™
Manuscripts

I, the Submitting Author has the right to grant and does grant on behalf of all authors of the Work (as defined in the below author licence), an exclusive licence and/or a non-exclusive licence for contributions from authors who are: i) UK Crown employees; ii) where BMJ has agreed a CC-BY licence shall apply, and/or iii) in accordance with the terms applicable for US Federal Government officers or employees acting as part of their official duties; on a worldwide, perpetual, irrevocable, royalty-free basis to BMJ Publishing Group Ltd ("BMJ") its licensees and where the relevant Journal is co-owned by BMJ to the co-owners of the Journal, to publish the Work in this journal and any other BMJ products and to exploit all rights, as set out in our licence.

The Submitting Author accepts and understands that any supply made under these terms is made by BMJ to the Submitting Author unless you are acting as an employee on behalf of your employer or a postgraduate student of an affiliated institution which is paying any applicable article publishing charge ("APC") for Open Access articles. Where the Submitting Author wishes to make the Work available on an Open Access basis (and intends to pay the relevant APC), the terms of reuse of such Open Access shall be governed by a Creative Commons licence – details of these licences and which Creative Commons licence will apply to this Work are set out in our licence referred to above.

Other than as permitted in any relevant BMJ Author's Self Archiving Policies, I confirm this Work has not been accepted for publication elsewhere, is not being considered for publication elsewhere and does not duplicate material already published. I confirm all authors consent to publication of this Work and authorise the granting of this licence.

For peer review only

The reliability and clinical relevance of outer retinal band length on OCT in retinal disease

Rene Cheung (BSc(Hons), BOptom, MOptom)[1,2], Angelica Ly (BOptom (Hons), GradCertOcTher, PhD)[1,2], Henrietta Wang (BOptom(Hons), BSc)[2], Michael Kalloniatis (BSc(Optom), MSc (Optom), PhD)[1,3] and Lisa Nivison-Smith (BSc (Hons), PhD) [1,2]

[1] School of Optometry and Vision Science; [2] Centre for Eye Health, University of New South Wales, Sydney, 2052, Australia; [3] School of Medicine (Optometry), Deakin University, Waurn Ponds, 3216, Victoria, Australia

Precis: Outer retinal bands on OCT can be measured reliably using magnitude estimation and significantly correlates with visual acuity. The lack of association to retinal disease highlights the importance of covariable adjustment when examining band disruptions.

Correspondence:

Dr Lisa Nivison-Smith

School of Optometry and Vision Science, UNSW Australia, Sydney, 2052, NSW, Australia.

Phone: Int +61 2 81150791

Fax: Int +61 2 81150799

E-mail: l.nivison-smith@unsw.edu.au

Key words: outer segments, optical coherence tomography, image analysis, retinal pigment epithelium, biomarker

For peer review only

The reliability and clinical relevance of outer retinal band length on OCT in retinal disease

ABSTRACT

Background: Hyperreflective outer retinal band (HORB) disruptions are associated with retinal disease, increasing with disease severity and progression. Reliable, easily implemented methods of assessing HORB disruptions are lacking. We assessed the reliability of using magnitude estimation to evaluate HORB length and determined its association with visual acuity and retinal disease.

Methods: HORB length (as a percentage of total B-scan length) was measured using magnitude estimation by two independent graders through foveal and non-foveal OCT B-scans through pathology in consecutive patients (n=600) referred to a secondary eyecare clinic in an observational study. Intra- and inter-grader reliability was calculated using a two-way random mixed-effects model with absolute agreement. Associations between HORB length, visual acuity, retinal disease and other clinical outcomes were considered statistically significant for *p*-values $\leq$ 0.05.

Results: Intragrader (ICC_{fovea}=0.81; ICC_{worst}=0.91) and intergrader (ICC_{fovea}=0.79; ICC_{worst}=0.75) agreement of HORB length was good to excellent. HORB length was significantly associated with age ($p<0.001$) and refractive error ($p<0.0001$-0.007) at all B-scan locations. Visual acuity ($p=0.002$), eccentricity ($p<0.001$) and B-scan length ($p<0.0001$) were associated with the primary outcome for foveal or non-foveal B-scans. Glaucoma was associated with HORB length on univariate analysis ($p=0.03$), however multivariate analysis revealed no significant association between HORB length and retinal disease (p=0.21-0.77).

Conclusion: HORB length is reliably assessed using magnitude estimation and may be useful as a surrogate biomarker of visual acuity. Several factors affect HORB length estimations, which may contribute to the lack of association to retinal disease and highlights the need for covariable adjustment when examining HORB disruptions.

Key points:

- Outer retinal disruptions have been correlated to retinal disease. However, it is unclear whether these can be measured reliably, and existing work is limited to highly selected patient populations.

- The length of undisrupted outer retinal bands on optical coherence tomography can be quantified reliably using magnitude estimation in an unselected patient population exhibiting a range of ocular pathology. Outer retinal band length is strongly associated with visual acuity, however, does not correlate with retinal disease after adjusting for covariable effects.

- Patient and imaging factors should be adjusted for in future analyses examining outer retinal disruptions in retinal disease to obtain a true understanding of associations to clinical outcomes.

INTRODUCTION

Outer retinal disruptions occur in a number of retinal diseases including retinal dystrophies,[1] pachychoroid spectrum disease,[2] diabetic retinopathy,[3] age-related macular degeneration (AMD),[4] and epiretinal membranes.[5] They are easily observed on optical coherence tomography (OCT) imaging as changes in hyperreflective, outer retinal band appearance and have been proposed as biomarkers of disease severity,[3, 6] recovery following treatment in neovascular AMD,[7] prognosis and visual acuity recovery in diabetic macula oedema.[8]

Many features have been explored to quantify changes in outer retinal band appearance on OCT with retinal disease including band continuity,[9] reflectivity[10] and thickness.[11] However, few studies have developed methods which emphasise easy translation and access in clinical settings. For example, some groups use custom software to perform assessments which are not readily available.[11, 12] Others evaluate features within a limited area around the foveal centre,[9, 13] which would be challenging to view by eye and therefore difficult to incorporate into qualitative evaluations of retinal images routinely performed in clinical practice. Studies also typically evaluate highly selected study populations without control groups,[10, 14] increasing the risk of selection bias and impacting the generalisability of findings.

The aim of this study was to assess outer retinal band appearance on OCT using magnitude estimation in an unbiased population. Magnitude estimation is an established, reliable technique for estimating sensory stimuli[15] including line length[16, 17] and produces measurements proportional to actual line length on average.[18, 19] It has been used to estimate optic nerve cup-to-disc ratio[20] and bulbar conjunctival redness[21] in clinical settings with good reliability. In this study we determined the reliability of measuring outer retinal band length using this method and assessed its association with clinical outcomes including visual acuity and retinal disease diagnosis in an unselected patient population.

METHODS

This study was approved by the Biomedical Human Research Ethics Advisory Panel of the University of New South Wales (Sydney, Australia). Prior informed written patient consent was obtained according to the Declaration of Helsinki.

Study location

Retrospective patient data was collected from the Centre for Eye Health (CFEH) Sydney, Australia. CFEH is an optometry-led referral centre that provides free diagnostic ocular imaging and disease management services to patients referred by optometrists and medical practitioners for additional assessment and collaborative care services with ophthalmologists. CFEH is a secondary eye care service attended by patients from the community who are suspected of a range of mild to moderate retinal diseases.[22]

For peer review only

Study population and screening

In total, 2039 unique consecutive patients seen at the CFEH between November 2nd, 2020, and January 18th, 2021 were screened for inclusion in the study. Patients were included in the study if they were: 1) referred from the community; 2) presented for an initial appointment during the study period and 3) were imaged by an OCT device at their visit. Patients were excluded if they were under 18 years of age, did not undergo a complete work-up involving diagnosis and management at CFEH, or if the OCT images in their record were below minimum scan quality requirements (Cirrus B-scans: Signal strength> 6; Spectralis scans: Quality > 15).

Data extraction

Date of birth, sex, visual acuity, dilation status, and diagnosis by CFEH clinicians were subsequently extracted from clinical records and patient reports using practice management software, Bp VIP.net (Best Practice Software). The extracted diagnosis was categorised by two investigators with a combined clinical experience of 20 years into the following mutually-exclusive categories: glaucoma disease, glaucoma suspect, optic nerve disease, macular disease, retinal disease, no ocular pathology, multiple diagnoses, no ocular pathology. All other diagnoses including anterior eye disease and vitreous anomalies were categorised as ‘other’. A combined category for outer retinal disease comprised of patients diagnosed with macular or retinal disease was created to further elucidate the association between outer retinal pathology and hyperreflective outer retinal band (HORB) length. Definitions used for diagnosis classification are described in **Table S1** and patients diagnosed with ageing changes or physiological variations were deemed to be without ocular pathology. The disease eye was selected as the study eye. If both eyes were eligible, the eye with greater disease severity was chosen.

OCT scan selection

For each patient, two B-scans were extracted from the study eye by an experienced clinician: one through the anatomically identified foveal pit, and the other through the worst area of retinal pathology at a non-foveal location . A non-foveal B-scan was chosen randomly if there was no retinal pathology. B-scans were extracted from the OCT volume with the highest axial resolution which were captured by either the Cirrus HD-OCT (Carl Zeiss Meditec, Dublin, CA, USA) or Spectralis OCT (Heidelberg Engineering, Inc., Heidelberg, Germany).[23] Details of different scan types extracted for final assessment for all study eyes are described in **Figure S1**.

After image extraction, all B-scans were de-identified and randomised to create two separate portable document files (PDF) of foveal or worst pathology B-scans. The process was then repeated to produce a second set of randomised images for grading to determine the reliability of estimations. Eccentricity of the non-foveal B-scan was calculated from its position relative to the foveal B-scan and interscan distance.

For peer review only

Magnitude estimation of outer retinal band length

Hyperreflective outer retinal band assessment

Magnitude estimation involves estimating the magnitude of a stimuli against an established reference value and is an established, reliable technique for estimating sensory stimuli[15] including line length.[16, 17] [18, 19] In this study, the total length of the OCT B-scan was used as the reference and the length of the HORB including the photoreceptor ellipsoid zone (EZ), interdigitation with RPE (IZ), and the RPE/Bruch's membrane complex (RPE)[24] was used as the stimuli (**Figure 1**). Final values were expressed as the estimated percentage of the scan which showed three distinct HORBs (i.e. EZ, IZ and RPE) relative to total length. For reference, in the healthy retina, HORB length would be estimated to be near 100% as they are continuous across an OCT B-scan aside from interferences from imaging artefacts, blood vessels or poor image quality and terminate adjacent to the optic disc.

Reliability of estimations

Intra-grader reliability was determined from comparison of repeated, blinded grading of the whole data set by investigator RC. Inter-grader reliability was determined from comparison of independent, blinded grading from investigator RC and HL for half the data set.

Statistical analysis

The sample size was determined from previous estimates of unique patients presenting for comprehensive examinations over a one-year period to ensure that the study population was representative of patients seen at the CFEH.[22] Intra- and inter-grader reliability of the magnitude estimation for HORB length was calculated using a two-way random mixed-effects model with absolute agreement. Intraclass correlations were interpreted using predefined criteria: <0.5 as poor, 0.5-0.75 as moderate, 0.75-0.9 as good, and >0.9 as excellent reliability.[25] The association between HORB length and diagnoses was determined using univariable and multivariable regression with p values less than 0.05 considered statistically significant. All statistical analyses were performed using SPSS (version 25; IBM, Armonk, New York, USA). All summary values are mean $\pm$ SD unless stated otherwise.

Patient and public involvement

Patients and the public were not involved in the design, conduct, reporting or dissemination plans of the research.

For peer review only

RESULTS

Study population

A total of 600 eligible patients were included in the study with 80.5% of patients (480/600) diagnosed or identified as at risk of developing ocular disease based on the final diagnosis. The most frequent diagnostic categories were glaucoma suspect (23.8%, 143/600) and macular disease (16.5%, 99/600). No ocular pathology was found in 19.5% of patients (117/600) (**Table S2**).

The mean age of all patients was 55.2 ± 15.2 years and 50.3% (302/600) were female. The mean best vision sphere and visual acuity (logMAR) of the study eye was -0.97 (2.93) Dioptres (D) and 0.05 (0.126), respectively (equivalent Snellen VA 6/22). Only age (*p<0.0001*) and visual acuity (*p=0.005*) were significantly different between patients with and without ocular pathology (**Table 1**).

Table 1: Characteristics of patients (N=600)

Demographic characteristics	No pathology	Ocular pathology	*p*
Female sex, % (n/N)	52.1 (61/117)	49.9 (241/483)	0.66
Age, mean years (SD)	49.4 (15.0)	56.6 (14.7)	**<0.001**
Study eye BVS, mean (SD)	-1.21 (2.9)	-0.92 (2.9)	0.34
Study eye VA (SD)	0.027 (0.09)	0.057 (0.13)	**0.005**
HORB length estimations			
Foveal B-scans	28.9 (28.2)	26.0 (27.3)	0.31
Non-foveal B-scans	22.2 (26.9)	18.7 (24.2)	0.17

Abbreviations: BVS, best vision sphere; VA, visual acuity; logMAR, logarithm of minimal angle resolution; SD, standard deviation.

HORB length

Examples of different HORB values for eyes with and without pathology are shown in **Figure 2**. The mean (SD) foveal HORB length in patients with and without ocular pathology was 26.0% (27.3) and 28.9% (28.2) respectively; for non-foveal B-scans, 18.7% (24.2) and 22.2% (26.9). There was no significant difference in HORB length for foveal and non-foveal B-scans between groups.

Reliability of HORB length estimations

Overall, the reliability of HORB length estimations by the same grader and between graders was good to excellent at foveal and non-foveal scan locations according to pre-defined criteria (ICC=0.75-0.91).[25] A sub-group analysis of reliability by OCT device and B-scan length also found that the reliability of HORB estimations for B-scans from Spectralis OCT (ICC=0.75-0.91) and B-scans 7-9mm in length (ICC=0.83-0.91) was good to excellent. For longer B-scans and those captured by Cirrus OCT, the intra and inter-grader reliability was at least moderate (ICC=0.53-0.91) (**Table 2**).

For peer review only

Table 2: Intraclass correlation results by device type and B-scan length

**Overall**	**N**		**Intra-rater reliability**	**N**	**Inter-rater reliability**
	600	*Foveal*	0.81	600	0.79
		Non-foveal	0.91		0.75
**Device**	**N**		**Intra-rater reliability**	**N**	**Inter-rater reliability**
Spectralis	151	*Foveal*	0.75	77	0.76
		Non-foveal	0.91	75	0.79
Cirrus	379	*Foveal*	0.83	185	0.82
		Non-foveal	0.91	187	0.72
**B-scan length**	**N**		**Intra-rater reliability**	**N**	**Inter-rater reliability**
5-7mm	123	*Foveal*	0.83	50	0.91
		Non-foveal	0.90	55	0.87
7-9mm	357	*Foveal*	0.81	175	0.88
		Non-foveal	0.91	169	0.68
9-11mm	34	*Foveal*	0.57	13	0.89
		Non-foveal	0.94	16	0.54
15-17mm	17	*Foveal*	0.76	12	0.80
		Non-foveal	0.95	11	0.53

Association between HORB length and retinal disease

For foveal B-scans, age (*p<0.001*), VA (*p<0.001*), BVS (*p=0.006*) and a diagnosis of glaucoma (*p=0.03*) were significantly associated with HORB length on univariate analysis for foveal B-scans. A diagnosis of macular disease or outer retinal disease showed near significant associations (p=0.06, p=0.06 respectively). When all variables which demonstrated a p<0.25 on univariable analysis were included into multivariable analysis, only age, VA and BVS remained significant (**Table 3**).
For non-foveal B-scans, age (*p<0.001*), VA (*p=0.001*), B-scan length (*p<0.001*), eccentricity (*p<0.001*), and glaucoma diagnosis (*p=0.03*) were significantly associated with HORB length on univariable analysis. A diagnosis of macular disease again showed a near significant association (p=0.06). When all variables demonstrating p<0.25 in univariable analysis were included in multivariable analysis, only age, BVS, B-scan length and eccentricity remained significant (**Table 3**).

Together, these results showed no significant association between HORB length and retinal disease. However, age and refractive error significantly affected HORB length at both the foveal and non-foveal locations. VA had a significant effect on foveal HORB length, while B-scan length and eccentricity significantly affected the HORB length of non-foveal B-scans.

For peer review only - http://bmjopen.bmj.com/site/about/guidelines.xhtml

Table 3: Association between patient/scan characteristics and HORB length

Univariable analysis	Foveal B-scans		Non-foveal B-scans	
	p	*Beta coefficient*†	*p*	*Beta coefficient*†
Age	**<0.001**	-0.21	**<0.001**	-0.33
Sex	0.64	-0.02	0.87	0.33
VA	**<0.001**	-0.20	**0.001**	-27.06
BVS	**0.006**	0.11	0.14	0.51
High-definition B-scan	0.30	3.97	0.40	2.92
B-scan length	0.20	-0.06	**<0.001**	-3.00
Eccentricity	-	-	**<0.001**	-3.16
Device type‡	0.55	-1.19	0.11	2.83
Diagnosis	0.70	0.15	0.34	0.34
**Diagnosis category**				
Glaucoma disease	**0.03**	-14.0	**0.03**	-12.85
Glaucoma suspect	0.81	0.83	0.38	-2.71
Macular disease	0.06	-7.00	0.06	-6.25
Retinal disease	0.40	-3.45	0.61	-1.88
Optic nerve disease	0.83	1.27	0.97	-0.23
Outer retinal disease	0.06	-4.61	0.24	-2.63
**Multiple linear regression analysis**§				
Age	**<0.0001**	-0.23	**<0.0001**	-0.25
VA	**0.002**	-0.14	0.11	-0.07
BVS	**<0.001**	0.17	**0.003**	0.13
B-scan length	0.27	-0.05	**<0.0001**	-0.18
Eccentricity	N/A	N/A	**<0.001**	-0.17
Glaucoma disease	0.34	-0.04	0.82	-0.01
Macular disease	0.44	-0.03	0.28	-0.05
Outer retinal disease	0.23	-0.05	0.23	-0.05

BVS = best vision sphere; VA = visual acuity; † Standardised; ‡ Spectralis or Cirrus device (excluding macular cube B-scans); § Includes all univariable analysis variables demonstrating p ≤ 0.25.[26]

DISCUSSION

This study found that HORB length could be assessed reliably from OCT B-scans using magnitude estimation. HORB length estimations were significantly associated with age and visual acuity, however they were not significantly associated with retinal disease after adjusting for covariables.

For peer review only

This points to importance of assessing the utility of outer retinal biomarkers in large, unselected patient populations and controlling for all confounders.

Reliability

HORB length can be reliably assessed using magnitude estimation across different OCT devices and scan protocols in patients with and without retinal pathology. Our results show that the reliability of estimating HORB length at the fovea was good to excellent across two major commercially available OCT devices and scan lengths of 9mm or less for an unselected population exhibiting a range of ocular pathology. Moreover, intra- and inter-rater reliability of HORB estimations was at least moderate for all devices or scan lengths tested at both foveal and non-foveal B-scan locations. At non-foveal locations, intra-rater reliability of magnitude estimations was excellent for both OCT devices and all scan lengths and but only moderate to good between graders, with the exception of B-scans captured by Spectralis OCT and B-scan lengths of 7mm or less. The greater axial resolution of Spectralis OCT B-scans (versus Cirrus OCT[23]) and increased difficulty of estimating HORB length across longer B-scan likely accounts for these findings.

Our results are in line with the few previous reports that found ELM and EZ integrity measurements reliable.[7, 27] Indeed, other applications of magnitude estimation in eye care such as optic nerve cup-to-disc ratio estimation [20] and bulbar conjunctival redness[21] grading, also show good reliability and are routinely used in clinical practice. Whilst categorical approaches have been used previously to evaluate outer retinal integrity, many do not report reliability and the continuous nature of magnitude estimations may be advantageous over these methods as visual assessment of clinical features using scales with smaller increments has been shown to improve inter and intra-rater reliability.[28, 29]

Association between outer retinal disruptions and clinical outcomes

We found visual acuity was significantly associated with foveal HORB length in an unselected patient population. This supports previous suggestions that outer retinal integrity could be a useful surrogate biomarker of visual acuity,[30] particularly in severe retinal disease such as retinitis pigmentosa where visual acuity measures become less repeatable or unobtainable.[31] HORB length was also significantly associated with age in both foveal and non-foveal scans, consistent with other reports,[10, 12] emphasising the need for age-correction when assessing image-based biomarkers.

We also identified refractive error, B-scan length and eccentricity as additional covariables affecting HORB length that have not been previously reported. Differences in cone density with axial length[32] may explain the association between refractive error and HORB length as band reflectivity varies with cone density[33] and affects band visibility. Lower cone densities are also found at greater eccentricities which may explain the negative correlation between HORB length and eccentricity in non-foveal scans. For B-scan length, the inverse association with HORB length may be due to greater averaging of disruptions over longer scan lengths. This effect was only significant for non-foveal scans, perhaps as foveal scans were arbitrarily selected and did not necessarily include outer retinal disruptions. Considering the number of studies emphasizing the prognostic potential of outer retinal disruptions[7, 8] and relationships to other clinical outcomes, the significant covariables identified in this study will be highly relevant to future validation of these biomarkers in retinal disease.

For peer review only

Other groups have reported significant reduction of ellipsoid zone intensity and interdigitation zone area in glaucomatous eyes compared to age-matched normal eyes.[34, 35] We also found that glaucoma disease was significantly associated with HORB length but this was not maintained when we adjusted for covariables in multivariate analysis. Similarly, outer retinal disease such as subretinal fluid[11] and early AMD[4] have been associated with compromised HORB integrity, yet we did not find a significant association after adjusting for covariables. The disparity between our study findings and previous evidence may stem from the recruitment of study participants from a non-tertiary care centre rather than hospital or ophthalmology clinic settings.[2, 5, 7, 11] where retinal disease patients are typically at more advanced stages and exhibit a higher degree of outer retinal disruption.[3, 5] Thus, examining magnitude estimation of HORB length in a population with more severe retinal disease may be useful to elucidate the strength of its association to disease.

Mean HORB length estimations were also lower than expected, particularly in the no ocular pathology group where we expected to see intact HORB at least throughout the foveal scan. The strong inverse correlation between age, visual acuity, B-scan length and eccentricity and HORB length suggests that these factors likely contributed to this result as the effect was sustained after adjusting for disease status. These findings again, emphasise the importance of adjusting for covariables when measuring outer retinal band biomarkers.

Strengths and limitations

A key strength to the study was using a consecutive recruitment strategy to identify an unselected patient population, which was more representative of individuals attending primary eye care practices. This therefore improves the generalisability of findings by enabling a true estimation of associations between HORB length, clinical outcomes and covariables. However, this also created limitations around sub-group sample sizes due to the low prevalence of retinal disease in patients referred to CFEH.[22] As such, further work to evaluate outer retinal disruptions in specific disease types and may benefit from oversampling target sub-groups.

A limitation of the study was that the ELM was not included as part of the HORB assessment even though ELM disruptions have also been correlated with retinal disease.[3, 13, 14] This structure was not included in the grading task as the intensity of the ELM is markedly reduced compared to the other bands and may be difficult to reliably detect by visual estimation. Given our findings, it may be worthwhile evaluating the impact of patient and imaging factors on ELM integrity in future investigations.

Another limitation was that all estimations were performed manually and no artificial (AI) intelligence-driven methods of quantifying HORB length were explored. While AI techniques have shown increasing promise for clinical applications,[36] the implementation of AI-driven tools in primary or tertiary eye care clinics is presently low with only 15.7% of ophthalmologists currently use AI-derived algorithms in daily practice.[37] Primary eye care practices are also known to have slow uptake of new clinical tools[38] with clinicians still reporting reservations about AI decision-support tools.[39] Thus, magnitude estimation was pursued as a technique that is highly accessible to clinical practices and requires no specialised equipment.

CONCLUSION

We show that outer retinal band length can be reliably assessed using magnitude estimation and may be useful as a surrogate biomarker of visual acuity. Importantly, this study is the first to assess

For peer review only

the reliability of measuring HORB integrity beyond the fovea and demonstrates that HORB length at non-foveal locations can be measured reliably using magnitude estimation. Several factors including age, refractive error, eccentricity and B-scan length affected HORB length estimations and highlights the importance of adjusting for covariables when evaluating HORB disruptions in retinal disease to ensure true associations are obtained.

ACKNOWLEDGEMENTS

The authors thank Natalie Eshow, Meri Galoyan and Matt Trinh for technical assistance and Helene Ly for grading support.

DISCLOSURES

Disclosure: LN-S, None; RC, None; HW, None; AL, None; MK, None

This work was presented as a conference abstract at the XXV Biennial Meeting of the International Society for Eye Research, February 19-23, 2023, Gold Coast, Queensland, Australia.

FUNDING

This work was supported by funding from the National Health and Medical Research Council (NHMRC #1174385, #1186915) and the Research Training Program scholarship. Guide Dogs NSW/ACT provides support for the Centre for Eye Health (the clinic from which patient data was obtained).

REFERENCES

1. Cetin EN, Parca O, Akkaya HS*, et al.* Association of retinal biomarkers and choroidal vascularity index on optical coherence tomography using binarization method in retinitis pigmentosa. Graefe's Archive for Clinical Experimental Ophthalmology 2020;**258**(1):23-30
2. Ojima Y, Tsujikawa A, Yamashiro K*, et al.* Restoration of outer segments of foveal photoreceptors after resolution of central serous chorioretinopathy. Jpn J Ophthalmol 2010;**54**(1):55-60 doi: 10.1007/s10384-009-0766-4[published Online First: Epub Date]|.
3. Jain A, Saxena S, Khanna VK*, et al.* Status of serum VEGF and ICAM-1 and its association with external limiting membrane and inner segment-outer segment junction disruption in type 2 diabetes mellitus. Molecular vision 2013;**19**:1760
4. Wu Z, Ayton LN, Guymer RH*, et al.* Relationship Between the Second Reflective Band on Optical Coherence Tomography and Multifocal Electroretinography in Age-Related Macular Degeneration. Investigative Ophthalmology & Visual Science 2013;**54**(4):2800-06 doi: 10.1167/iovs.13-11613[published Online First: Epub Date]|.
5. Schumann RG, Compera D, Schaumberger MM*, et al.* Epiretinal membrane characteristics correlate with photoreceptor layer defects in lamellar macular holes and macular pseudoholes. Retina 2015;**35**(4):727-35 doi: 10.1097/IAE.0000000000000375[published Online First: Epub Date]|.
6. Das R, Spence G, Hogg RE*, et al.* Disorganization of Inner Retina and Outer Retinal Morphology in Diabetic Macular Edema. JAMA Ophthalmol 2018;**136**(2):202-08 doi: 10.1001/jamaophthalmol.2017.6256[published Online First: Epub Date]|.

For peer review only

7. Oishi A, Shimozono M, Mandai M*, et al.* Recovery of photoreceptor outer segments after anti-VEGF therapy for age-related macular degeneration. Graefe's archive for clinical and experimental ophthalmology 2013;**251**(2):435-40
8. Muftuoglu IK, Mendoza N, Gaber R*, et al.* Integrity of outer retinal layers after resolution of central involved diabetic macular edema. Retina (Philadelphia, Pa.) 2017;**37**(11):2015
9. Sundaram V, Wilde C, Aboshiha J*, et al.* Retinal structure and function in achromatopsia: implications for gene therapy. Ophthalmology 2014;**121**(1):234-45 doi: 10.1016/j.ophtha.2013.08.017[published Online First: Epub Date]|.
10. Gin TJ, Wu Z, Chew SKH*, et al.* Quantitative Analysis of the Ellipsoid Zone Intensity in Phenotypic Variations of Intermediate Age-Related Macular Degeneration. Investigative Ophthalmology & Visual Science 2017;**58**(4):2079-86 doi: 10.1167/iovs.16-20105[published Online First: Epub Date]|.
11. Hood DC, Zhang X, Ramachandran R*, et al.* The inner segment/outer segment border seen on optical coherence tomography is less intense in patients with diminished cone function. Investigative ophthalmology & visual science 2011;**52**(13):9703-09
12. Arepalli S, Traboulsi EI, Ehlers JP. Ellipsoid zone mapping and outer retinal assessment in Stargardt disease. Retina 2018;**38**(7):1427
13. Nakamura T, Ueda-Consolvo T, Oiwake T*, et al.* Correlation between outer retinal layer thickness and cone density in patients with resolved central serous chorioretinopathy. Graefe's Archive for Clinical and Experimental Ophthalmology 2016;**254**(12):2347-54
14. Wu Z, Ayton LN, Guymer RH*, et al.* Second reflective band intensity in age-related macular degeneration. Ophthalmology 2013;**120**(6):1307-08. e1
15. Gescheider GA. Psychophysical scaling. Annual review of psychology 1988;**39**(1):169-200
16. Verrillo RT. Stability of line-length estimates using the method of absolute magnitude estimation. Perception Psychophysics 1983;**33**(3):261-65
17. Gescheider GA, Hughson BA. Stimulus context and absolute magnitude estimation: A study of individual differences. Perception & Psychophysics 1991;**50**(1):45-57
18. Stevens SS, Marks LE. *Psychophysics: Introduction to its perceptual, neural, and social prospects*: Routledge, 2017.
19. Stevens SS. On the psychophysical law. Psychol Rev 1957;**64**(3):153-81 doi: 10.1037/h0046162[published Online First: Epub Date]|.
20. Feuer WJ, Parrish II RK, Schiffman JC*, et al.* The Ocular Hypertension Treatment Study: reproducibility of cup/disk ratio measurements over time at an optic disc reading center. American journal of ophthalmology 2002;**133**(1):19-28
21. Chong T, Simpson T, Fonn D. The repeatability of discrete and continuous anterior segment grading scales. Optometry and Vision Science 2000;**77**(5):244-51
22. Wang H, Kalloniatis M. Clinical outcomes of the Centre for Eye Health: an intra-professional optometry-led collaborative eye care clinic in Australia. Clinical Experimental Optometry 2021:1-10
23. Leite MT RH, Zangwill LM, Weinreb RN, Medeiros FA. Comparison of the diagnostic accuracies of the Spectralis, Cirrus, and RTVue optical coherence tomography devices in glaucoma. Ophthalmology 2011;**118**(7):1334-9
24. Staurenghi G, Sadda S, Chakravarthy U*, et al.* Proposed lexicon for anatomic landmarks in normal posterior segment spectral-domain optical coherence tomography: the IN• OCT consensus. Ophthalmology 2014;**121**(8):1572-78
25. Koo TK, Li MY. A Guideline of Selecting and Reporting Intraclass Correlation Coefficients for Reliability Research. J Chiropr Med 2016;**15**(2):155-63 doi: 10.1016/j.jcm.2016.02.012[published Online First: Epub Date]|.
26. Bursac Z, Gauss CH, Williams DK*, et al.* Purposeful selection of variables in logistic regression. Source code for biology and medicine 2008;**3**(1):1-8

For peer review only

27. Reibaldi M, Parravano M, Varano M*, et al.* Foveal microstructure and functional parameters in lamellar macular hole. American journal of ophthalmology 2012;**154**(6):974-80. e1
28. Fowler PV, Al-Ani AH, Thompson JM. Comparison of reliability of categorical and continuous scales for radiographic assessments of bone infill following secondary alveolar bone grafting. The Cleft Palate-Craniofacial Journal 2018;**55**(2):269-75
29. Masmali AM, Murphy PJ, Purslow C. Development of a new grading scale for tear ferning. Contact Lens and Anterior Eye 2014;**37**(3):178-84
30. Sousa K, Fernandes T, Gentil R*, et al.* Outer retinal layers as predictors of visual acuity in retinitis pigmentosa: a cross-sectional study. Graefe's Archive for Clinical and Experimental Ophthalmology 2019;**257**:265-71
31. Kiser AK, Mladenovich D, Eshraghi F*, et al.* Reliability and consistency of visual acuity and contrast sensitivity measures in advanced eye disease. Optometry and vision science 2005;**82**(11):946-54
32. Chui TYP, Song H, Burns SA. Individual variations in human cone photoreceptor packing density: variations with refractive error. Investigative ophthalmology & visual science 2008;**49**(10):4679-87
33. Flores M, Debellemanière G, Bully A*, et al.* Reflectivity of the outer retina on spectral-domain optical coherence tomography as a predictor of photoreceptor cone density. American Journal of Ophthalmology 2015;**160**(3):588-95. e2
34. Ha A, Kim YK, Jeoung JW*, et al.* Ellipsoid zone change according to glaucoma stage advancement. American journal of ophthalmology 2018;**192**:1-9
35. Ha A, Kim YK, Lee J*, et al.* Interdigitation Zone Change According to Glaucoma-Stage Advancement. Invest Ophthalmol Vis Sci 2020;**61**(4):20 doi: 10.1167/iovs.61.4.20[published Online First: Epub Date]|.
36. Keenan TD, Clemons TE, Domalpally A*, et al.* Retinal Specialist versus Artificial Intelligence Detection of Retinal Fluid from OCT: Age-Related Eye Disease Study 2: 10-Year Follow-On Study. Ophthalmology 2021;**128**(1):100-09
37. Scheetz J, Koklanis K, McGuinness M*, et al.* Gaze behaviour and accuracy among novice and glaucoma specialist orthoptists during optic disc examination: A cross sectional study. Australian Orthoptic Journal 2019;**51**:17-24
38. Cheung R, Ho S, Ly A. Optometrists' attitudes toward using OCT angiography lag behind other retinal imaging types. Ophthalmic and Physiological Optics 2023
39. Safi S, Thiessen T, Schmailzl KJ. Acceptance and resistance of new digital technologies in medicine: qualitative study. JMIR research protocols 2018;**7**(12):e11072

For peer review only

FIGURES

Figure 1: *(A)* High definition, Cirrus B-scan of a healthy fovea with *(B)* the hyperreflective outer retinal bands (HORB) assessed for outer retinal integrity: ellipsoid zone (red), interdigitation zone (blue) and RPE/ Bruch's membrane complex (green). The external limiting membrane (white arrows, panel B) was not included in our definition of HORB.

Figure 2: Examples of foveal (left) and worst (right) B-scans for eyes with *(A-B)* no retinal pathology, *(C-D)* neovascular AMD, *(E-F)* central serous chorioretinopathy and *(G-H)* multifocal pattern dystrophy. Estimation of HORB length is indicated in the top right-hand corner of each panel.

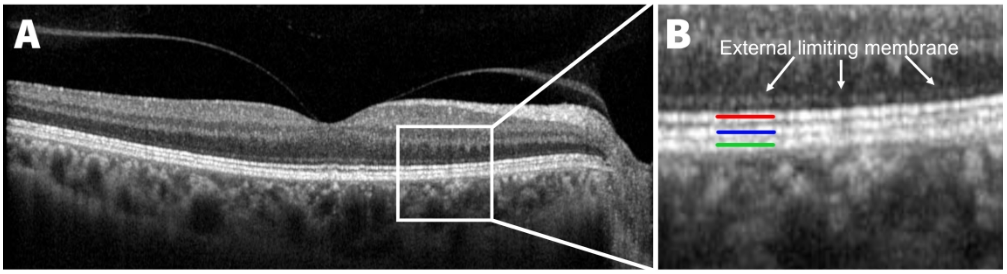

1309x363mm (72 x 72 DPI)

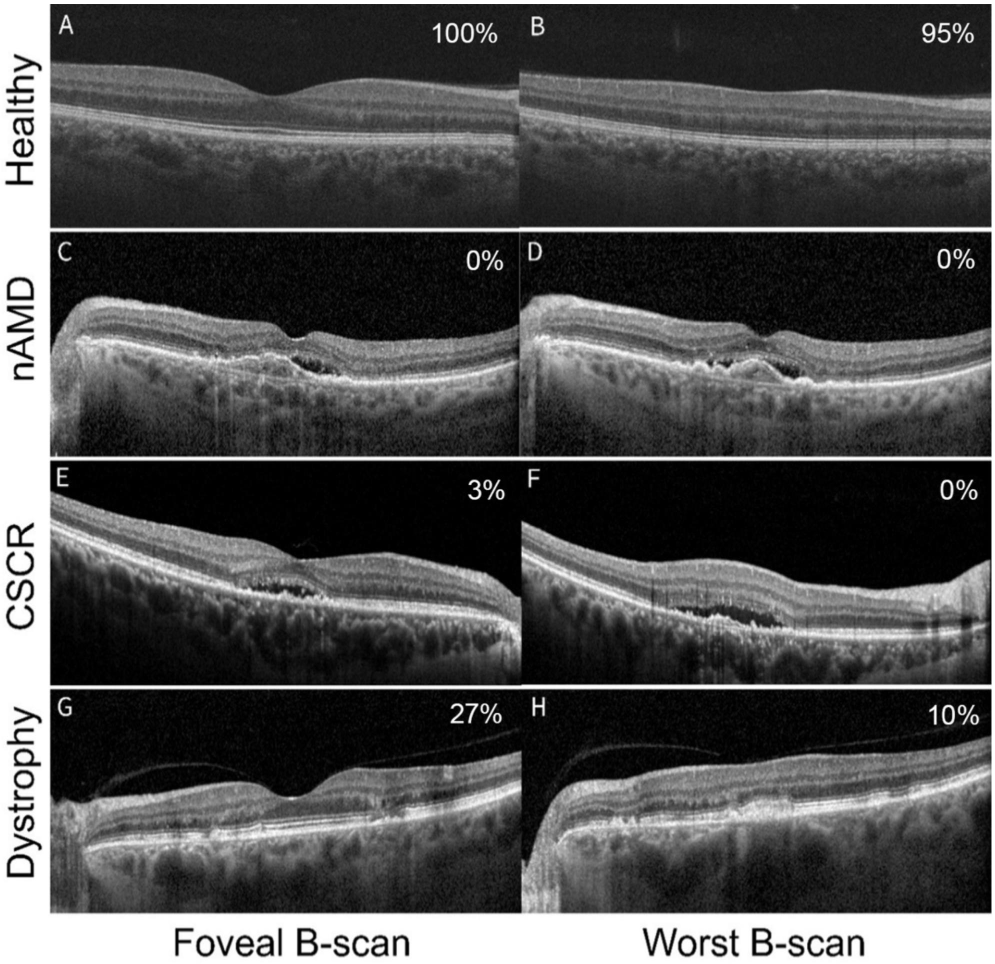

1096x1060mm (72 x 72 DPI)

Supplementary Material

Methods – OCT B-scan grading instructions

OCT B-scans were graded using the following instructions: ‘*Not counting the area directly under the optic nerve (if your image contains an optic nerve), what percentage of the horizontal length of the scan shows three complete and continuous hyperreflective lines in the retinal layers between the myoid zone and choriocapillaris?*’ All images were reviewed in full-screen mode at 100% magnification, maximal brightness and standard contrast settings (Asus Vivobook S15).

Table S1: Diagnosis classification

Diagnosis category	Criteria for inclusion
Glaucoma disease	Glaucoma diagnosis recorded in clinical record
Glaucoma suspect	• Glaucoma risk requiring further work-up due to historical risk factors and/or detection of structural and/or functional signs suggestive of glaucoma.<br>• Narrow angles requiring further monitoring
Optic nerve disease	Non-glaucomatous optic nerve pathology
Macular disease	• AMD<br>• Pachychoroid spectrum disease<br>• Other macular pathology e.g. retinal dystrophy, epiretinal membranes<br>• Non-specific macular pathology e.g. isolated pigmented disruption or drusen
Retinal disease	• Diabetic retinopathy<br>• Myopia-related complications<br>• Extra-macular retinal pathology unrelated to myopia or diabetes
Other	• Corneal degenerations<br>• Visual symptoms or headaches unexplained by clinical findings<br>• Pigmented lesion<br>• Anterior eye disease including dry eye, conjunctival lesions or other pathology unrelated to the cornea<br>• Vitreous anomalies
Multiple diagnoses	Diagnosed with multiple conditions in different categories
No ocular pathology	No ocular pathology noted in clinician report including druplets, cataracts, refractive errors and physiological cupping or nerve crowding.
Outer retinal disease	Diagnosis of macular and/or retinal disease

For peer review only

Table S2: Patient diagnoses

Diagnostic category	(%, n/N)
Glaucoma suspect	23.8 (143/600)
Macular disease	16.5 (99/600)
Retinal disease	12.0 (72/600)
Multiple diagnoses	10.8 (65/600)
Other diagnoses	9.3 (56/600)
Optic nerve disease	4.5 (27/600)
Glaucoma disease	3.5 (21/600)
No ocular pathology	19.5 (117/600)

Figure S1: pie chart of OCT scan patterns from which B-scans were extracted. *Other scan types include B-scans from Spectralis 20x20 scan patterns and single line B-scans.

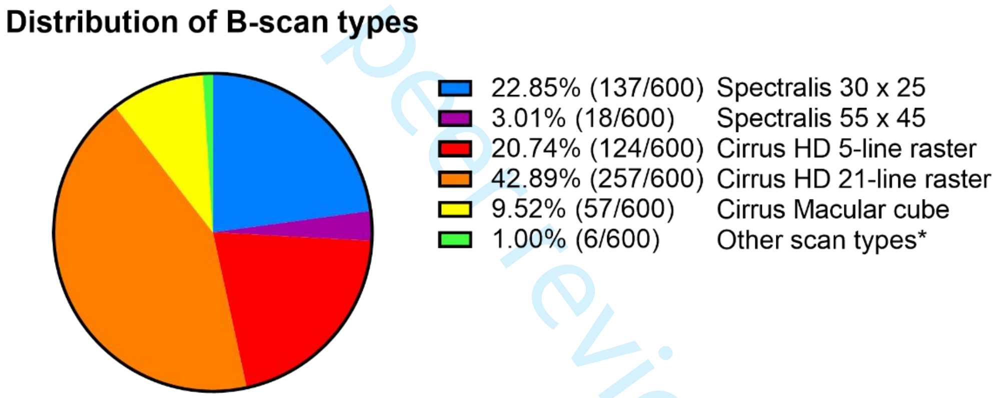

For peer review only

STROBE Statement—Checklist of items that should be included in reports of ***cross-sectional studies***

	Item No	Recommendation	Page No
**Title and abstract**	1	(*a*) Indicate the study's design with a commonly used term in the title or the abstract	2
		(*b*) Provide in the abstract an informative and balanced summary of what was done and what was found	2
**Introduction**			
Background/rationale	2	Explain the scientific background and rationale for the investigation being reported	3
Objectives	3	State specific objectives, including any prespecified hypotheses	3
**Methods**			
Study design	4	Present key elements of study design early in the paper	3
Setting	5	Describe the setting, locations, and relevant dates, including periods of recruitment, exposure, follow-up, and data collection	3
Participants	6	(*a*) Give the eligibility criteria, and the sources and methods of selection of participants	3-4
Variables	7	Clearly define all outcomes, exposures, predictors, potential confounders, and effect modifiers. Give diagnostic criteria, if applicable	4-5
Data sources/ measurement	8*	For each variable of interest, give sources of data and details of methods of assessment (measurement). Describe comparability of assessment methods if there is more than one group	4-5
Bias	9	Describe any efforts to address potential sources of bias	3
Study size	10	Explain how the study size was arrived at	5
Quantitative variables	11	Explain how quantitative variables were handled in the analyses. If applicable, describe which groupings were chosen and why	5
Statistical methods	12	(*a*) Describe all statistical methods, including those used to control for confounding	5
		(*b*) Describe any methods used to examine subgroups and interactions	5
		(*c*) Explain how missing data were addressed	3-4
		(*d*) If applicable, describe analytical methods taking account of sampling strategy	
		(*e*) Describe any sensitivity analyses	4
**Results**			
Participants	13*	(a) Report numbers of individuals at each stage of study—eg numbers potentially eligible, examined for eligibility, confirmed eligible, included in the study, completing follow-up, and analysed	3 & 5
		(b) Give reasons for non-participation at each stage	4-4
		(c) Consider use of a flow diagram	N/A
Descriptive data	14*	(a) Give characteristics of study participants (eg demographic, clinical, social) and information on exposures and potential confounders	5-6
		(b) Indicate number of participants with missing data for each variable of interest	N/A
Outcome data	15*	Report numbers of outcome events or summary measures	6-7
6Main results	16	(*a*) Give unadjusted estimates and, if applicable, confounder-adjusted estimates and their precision (eg, 95% confidence interval). Make clear which confounders were adjusted for and why they were included	7-8

For peer review only - http://bmjopen.bmj.com/site/about/guidelines.xhtml

		(*b*) Report category boundaries when continuous variables were categorized	6
		(*c*) If relevant, consider translating estimates of relative risk into absolute risk for a meaningful time period	N/A
Other analyses	17	Report other analyses done—eg analyses of subgroups and interactions, and sensitivity analyses	6-7
**Discussion**			
Key results	18	Summarise key results with reference to study objectives	8
Limitations	19	Discuss limitations of the study, taking into account sources of potential bias or imprecision. Discuss both direction and magnitude of any potential bias	9
Interpretation	20	Give a cautious overall interpretation of results considering objectives, limitations, multiplicity of analyses, results from similar studies, and other relevant evidence	8-9
Generalisability	21	Discuss the generalisability (external validity) of the study results	8
**Other information**			
Funding	22	Give the source of funding and the role of the funders for the present study and, if applicable, for the original study on which the present article is based	10

*Give information separately for exposed and unexposed groups.

Note: An Explanation and Elaboration article discusses each checklist item and gives methodological background and published examples of transparent reporting. The STROBE checklist is best used in conjunction with this article (freely available on the Web sites of PLoS Medicine at http://www.plosmedicine.org/, Annals of Internal Medicine at http://www.annals.org/, and Epidemiology at http://www.epidem.com/). Information on the STROBE Initiative is available at www.strobe-statement.org.

For peer review only

For peer review only - http://bmjopen.bmj.com/site/about/guidelines.xhtml

Evaluating the clinical relevance and reliability of outer retinal band length on optical coherence tomography in retinal disease: a cross-sectional study

Journal:	*BMJ Open*
Manuscript ID	bmjopen-2023-077874.R1
Article Type:	Original research
Date Submitted by the Author:	08-Nov-2023
Complete List of Authors:	Cheung, Rene; University of New South Wales Faculty of Science, School of Optometry and Vision Science; UNSW Centre for Eye Health<br>Ly, Angelica; University of New South Wales Faculty of Science, School of Optometry and Vision Science; UNSW Centre for Eye Health<br>Wang, Henrietta; UNSW Centre for Eye Health; University of New South Wales, Optometry and Vision Science<br>Kalloniatis, Michael; University of New South Wales, School of Optometry and Vision Science; Deakin University<br>Nivison-Smith, Lisa; University of New South Wales Faculty of Science, School of Optometry and Vision Science
**Primary Subject Heading**:	Ophthalmology
Secondary Subject Heading:	Research methods
Keywords:	Medical retina < OPHTHALMOLOGY, Diagnostic Imaging, Primary Health Care, Retrospective Studies

SCHOLARONE™
Manuscripts

I, the Submitting Author has the right to grant and does grant on behalf of all authors of the Work (as defined in the below author licence), an exclusive licence and/or a non-exclusive licence for contributions from authors who are: i) UK Crown employees; ii) where BMJ has agreed a CC-BY licence shall apply, and/or iii) in accordance with the terms applicable for US Federal Government officers or employees acting as part of their official duties; on a worldwide, perpetual, irrevocable, royalty-free basis to BMJ Publishing Group Ltd ("BMJ") its licensees and where the relevant Journal is co-owned by BMJ to the co-owners of the Journal, to publish the Work in this journal and any other BMJ products and to exploit all rights, as set out in our licence.

The Submitting Author accepts and understands that any supply made under these terms is made by BMJ to the Submitting Author unless you are acting as an employee on behalf of your employer or a postgraduate student of an affiliated institution which is paying any applicable article publishing charge ("APC") for Open Access articles. Where the Submitting Author wishes to make the Work available on an Open Access basis (and intends to pay the relevant APC), the terms of reuse of such Open Access shall be governed by a Creative Commons licence – details of these licences and which Creative Commons licence will apply to this Work are set out in our licence referred to above.

Other than as permitted in any relevant BMJ Author's Self Archiving Policies, I confirm this Work has not been accepted for publication elsewhere, is not being considered for publication elsewhere and does not duplicate material already published. I confirm all authors consent to publication of this Work and authorise the granting of this licence.

For peer review only

Evaluating the clinical relevance and reliability of outer retinal band length on optical coherence tomography in retinal disease: a cross-sectional study

Rene Cheung (BSc(Hons), BOptom, MOptom)[1,2], Angelica Ly (BOptom (Hons), GradCertOcTher, PhD)[1,2], Henrietta Wang (BOptom(Hons), BSc)[2], Michael Kalloniatis (BSc(Optom), MSc (Optom), PhD)[1,3] and Lisa Nivison-Smith (BSc (Hons), PhD) [1,2]

[1] School of Optometry and Vision Science; [2] Centre for Eye Health, University of New South Wales, Sydney, 2052, Australia; [3] School of Medicine (Optometry), Deakin University, Waurn Ponds, 3216, Victoria, Australia

Correspondence to:

Dr Lisa Nivison-Smith

School of Optometry and Vision Science, UNSW Australia, Sydney, 2052, NSW, Australia.

Phone: Int +61 2 81150791

Fax: Int +61 2 81150799

E-mail: l.nivison-smith@unsw.edu.au

Keywords: outer segments, optical coherence tomography, image analysis, retinal pigment epithelium, biomarker

ABSTRACT

Objectives: Hyperreflective outer retinal band (HORB) disruptions are reported across a range of retinal disease, yet a reliable, easily implemented assessment method and thorough evaluation of their association to retinal disease is lacking. The purpose of the study was to assess the reliability of using magnitude estimation to evaluate HORB length and determine its association to visual acuity and retinal disease.

Design: Cross-sectional, retrospective study.

Setting: Patients attending a secondary eye care clinic in Sydney, Australia.

Participants: 2039 unique consecutive patients were screened for inclusion between November 2nd, 2020, and January 18th,2021, and 600 were included in the study population. Patients were included if they were referred from primary care, presented for an initial, comprehensive eye examination during the study period, imaged with optical coherence tomography (OCT) during their visit and over 18 years of age.

For peer review only

Primary outcome: Reliability of HORB length estimations and associations to clinical outcomes.

Results: Intragrader (ICC_{fovea}=0.81; ICC_{worst}=0.91) and intergrader (ICC_{fovea}=0.78-0.79; ICC_{worst}=0.75-0.88) agreement of HORB length was good to excellent. HORB length was significantly associated with age (*p<0.001,* β=-0.22, -0.24) and refractive error (*p<0.001,* β=0.12-0.16) at all B-scan locations. Visual acuity (*p=0.001*, β=-0.13) was associated with the primary outcome for foveal B-scans and eccentricity (*p=0.002*, β=-0.13) and device type (*p=0.002*, β=0.13) for non-foveal B-scans. Glaucoma was associated with HORB length on univariate analysis (*p=0*.05-0.06, β=-0.08), however, multivariate analysis revealed no significant association between HORB length and retinal disease.

Conclusion: HORB length is reliably assessed using magnitude estimation and may be useful as a surrogate biomarker of visual acuity. Several factors affect HORB length estimations, which may contribute to the lack of association to retinal disease and highlights the need for covariable adjustment when examining HORB disruptions.

Strengths and limitations of this study

- An unselected population was obtained through consecutive recruitment, allowing a robust evaluation of associations between HORB length and retinal disease that is more generalisable to primary eye care settings.
- Magnitude estimation was used to assess outer retinal integrity, which is a reliable psychophysical technique that can be applied to OCT imaging without specialised equipment.
- The consecutive recruitment design limited sub-analysis of associations for specific categories of retinal disease.
- Despite previous correlations of external limiting membrane (ELM) disruptions to retinal disease, it was not included in HORB analysis due to its relatively weak intensity.
- Outer retinal band length was not measured using computerised methods.

INTRODUCTION

Outer retinal disruptions occur in a number of retinal diseases including retinal dystrophies,[1] pachychoroid spectrum disease,[2] diabetic retinopathy,[3] age-related macular degeneration (AMD),[4] and epiretinal membranes.[5] They are easily observed on optical coherence tomography (OCT) imaging as changes in hyperreflective, outer retinal band appearance and have been proposed as biomarkers of disease severity,[3, 6] recovery following treatment in neovascular AMD,[7] prognosis and visual acuity recovery in diabetic macula oedema.[8]

Many features have been explored to quantify changes in outer retinal band appearance on OCT with retinal disease including band continuity,[9] reflectivity[10] and thickness.[11] However, few studies have developed methods which emphasise easy translation and access in clinical settings. For example, some groups use custom software to perform assessments which are not readily available.[11, 12] Others evaluate features within a limited area around the foveal centre,[9, 13] which would be challenging to view by eye and therefore difficult to incorporate into qualitative evaluations of retinal images routinely performed in clinical practice. Studies also typically evaluate highly selected study populations without control groups,[10, 14] increasing the risk of selection bias and impacting the generalisability of findings.

For peer review only - http://bmjopen.bmj.com/site/about/guidelines.xhtml

The aim of this study was to assess outer retinal band appearance on OCT using magnitude estimation in an unbiased population. Magnitude estimation is an established, reliable technique for estimating sensory stimuli[15] including line length[16, 17] and produces measurements proportional to actual line length on average.[18, 19] It has been used to estimate optic nerve cup-to-disc ratio[20] and bulbar conjunctival redness[21] in clinical settings with good reliability. In this study we determined the reliability of measuring outer retinal band length using this method and assessed its association with clinical outcomes including visual acuity and retinal disease diagnosis in an unselected patient population.

METHODS

Study design and ethics approval

This retrospective, cross-sectional study was approved by the Biomedical Human Research Ethics Advisory Panel of the University of New South Wales (Sydney, Australia) (HC190746; June 2020). Written patient consent for use of medical data for research is obtained at the initial consultation at the Centre for Eye Health as standard protocol in line with the Declaration of Helsinki, which is made available for retrospective analysis.

Study setting

Retrospective patient data was collected from the Centre for Eye Health (CFEH) Sydney, Australia. CFEH is an optometry-led referral centre that provides free diagnostic ocular imaging and disease management services to patients referred by optometrists and medical practitioners for additional assessment and collaborative care services with ophthalmologists. CFEH is a secondary eye care service attended by patients from the community who are suspected of a range of mild to moderate retinal diseases.[22]

Study population and screening

In total, 2039 unique consecutive patients seen at the CFEH between November 2nd, 2020, and January 18th,2021 were screened for inclusion in the study. Patients were included in the study if they were: 1) referred from the community; 2) presented for an initial appointment during the study period and 3) were imaged by an OCT device at their visit. Patients were excluded if they were under 18 years of age, did not undergo a complete work-up involving diagnosis and management at CFEH, or if the OCT images in their record were below minimum scan quality requirements (Cirrus B-scans: Signal strength> 6; Spectralis scans: Quality > 15).

Data extraction

Date of birth, sex, visual acuity, dilation status, and diagnosis by CFEH clinicians were subsequently extracted from clinical records and patient reports using practice management software, Bp VIP.net (Best Practice Software). The extracted diagnosis was categorised by two investigators with a combined clinical experience of 20 years into the following mutually exclusive categories: glaucoma disease, glaucoma suspect, optic nerve disease, macular disease, retinal disease, no ocular pathology, multiple diagnoses, no ocular pathology. All other diagnoses including anterior eye disease and

For peer review only - http://bmjopen.bmj.com/site/about/guidelines.xhtml

vitreous anomalies were categorised as 'other'. A combined category for outer retinal disease comprised of patients diagnosed with macular or retinal disease was created to further elucidate the association between outer retinal pathology and hyperreflective outer retinal band (HORB) length. Definitions used for diagnosis classification are described in **Table S1** and patients diagnosed with ageing changes or physiological variations were deemed to be without ocular pathology. The disease eye was selected as the study eye. If both eyes were eligible, the eye with greater disease severity was chosen.

OCT scan selection

For each patient, two B-scans were extracted from the study eye by an experienced clinician: one through the anatomically identified foveal pit, and the other through the worst area of retinal pathology at a non-foveal location. A non-foveal B-scan was chosen randomly if there was no retinal pathology. B-scans were extracted from the OCT volume with the highest axial resolution which were captured by either the Cirrus HD-OCT (Carl Zeiss Meditec, Dublin, CA, USA) or Spectralis OCT (Heidelberg Engineering, Inc., Heidelberg, Germany).[23] Details of different scan types extracted for final assessment for all study eyes are described in **Figure S1**.

After image extraction, all B-scans were de-identified and randomised to create two separate portable document files (PDF) of foveal or worst pathology B-scans. The process was then repeated to produce a second set of randomised images for grading to determine the reliability of estimations. Eccentricity of the non-foveal B-scan was calculated from its position relative to the foveal B-scan and interscan distance.

Magnitude estimation of outer retinal band length

Hyperreflective outer retinal band assessment

Magnitude estimation involves estimating the magnitude of a stimuli against a reference value and is an established, reliable technique for estimating sensory stimuli[15] including line length.[16, 17] [18, 19] This approach was used as the magnitude estimation technique requires subjects to assess relative values (i.e. ratios) rather than actual length or size of stimuli in real units. Our approach is also consistent with other applications of magnitude estimation for evaluating ocular structures such as using optic disc size as the reference for cup-disc ratio estimations ratio[20] or the width of a large vein at the disc margin to estimate drusen size in age-related macular macular degeneration.[24] In this study, the total length of the OCT B-scan was used as the reference and the length of the HORB including the photoreceptor ellipsoid zone (EZ), interdigitation with RPE (IZ), and the RPE/Bruch's membrane complex (RPE)[25] was used as the stimuli (**Figure 1**). Final values were expressed as the estimated percentage of the scan which showed three distinct HORBs (i.e. EZ, IZ and RPE) relative to total length. For reference, in the healthy retina, HORB length would be estimated to be near 100% as they are continuous across an OCT B-scan aside from interferences from imaging artefacts, blood vessels or poor image quality and terminate adjacent to the optic disc. An OCT B-scan of a healthy eye with visibility of all three outer retinal bands verified by a senior research clinician of more than 40 years (author MK) was shown to all graders before commencing the task. All graders were clinicians experienced in OCT interpretation to ensure that gradings were not biased by image artefacts such as

For peer review only - http://bmjopen.bmj.com/site/about/guidelines.xhtml

blood vessel shadowing and only images meeting minimum quality control criteria were included to control for noise.

Reliability of estimations

Intra-grader reliability was determined by assessing the initial and repeat grading performed by one investigator (RC) for all patients (n=600). Repeat gradings were performed one week after the initial gradings. Inter-grader reliability was determined by comparing independent gradings from RC (grader 1) against HL (grader 2) and AD (grader 3) for half the data set (n=300). Grader 2 and 3 completed all gradings within a one-month period and both graders were blinded to the image randomization process and other patient identifiers.

Statistical analysis

We estimated that a sample size of at least 593 participants would be required to detect a 9% difference in band length between diseased versus non-diseased patients with 85% power to a 90% confidence level. The effect size was determined as the length over which existing grading methods assume that outer retinal bands can be visually discriminated as ‘absent’ or ‘present’ (the central 1mm) over the length of a B-scan (maximum 11mm). Intra- and inter-grader reliability of the magnitude estimation for HORB length was calculated using a two-way mixed-effects model with absolute agreement. A two-way mixed-effects model was selected as reliability will be assessed using gradings from selected raters rather than randomly drawn raters from a larger population of raters, for which one or two-way random-effects is more appropriate. Intraclass correlation (ICC) of a single rater was calculated as this reflects typical approaches to estimating clinical features in practice by a single clinician. ICC was interpreted using predefined criteria: <0.5 as poor, 0.5-0.75 as moderate, 0.75-0.9 as good, and >0.9 as excellent reliability.[26] The association between HORB length measured by grader 1 and diagnoses was determined using univariable and multivariable regression with *p* values less than 0.05 considered statistically significant. All statistical analyses were performed using SPSS (version 25; IBM, Armonk, New York, USA). All summary values are mean ± SD unless stated otherwise.

Patient and public involvement

None.

RESULTS

Study population

A total of 2039 patients were screened for inclusion into the study. Patients were excluded if medical consent for research was declined (n=317), they were under 18 years of age (n=37), attended for a follow-up rather than initial appointment (n=1033), did not have OCT performed at visit (n=28) or a complete work-up in clinical record (n=4), were referred from tertiary eye care settings (n=12) or did not have a final diagnosis in their record (n=8). The final sample size of 600 participants was sufficient to detect minimum disruptions in band length of 9%. In total, 80.5% of patients (480/600) were

For peer review only - http://bmjopen.bmj.com/site/about/guidelines.xhtml

diagnosed or identified as at risk of developing ocular disease based on the final diagnosis. The most frequent diagnostic categories were glaucoma suspect (23.8%, 143/600) and macular disease (16.5%, 99/600). No ocular pathology was found in 19.5% of patients (117/600) (**Table S2**).

The mean age of all patients was 55.2 ± 15.2 years and 50.3% (302/600) were female. The mean best vision sphere and visual acuity (logMAR) of the study eye was -0.97 (2.93) Dioptres (D) and 0.05 (0.126), respectively (equivalent Snellen VA 6/22). Only age (*p<0.0001*) and visual acuity (*p=0.005*) were significantly different between patients with and without ocular pathology (**Table 1**).

Table 1. Patient characteristics (N=600)

**Demographic characteristics**	**No pathology**	**Ocular pathology**	***p***
Female sex, % (n/N)	52.1 (61/117)	49.9 (241/483)	0.66
Age, mean years (SD)	49.4 (15.0)	56.6 (14.7)	**<0.001**
Study eye BVS, mean (SD)	-1.21 (2.9)	-0.92 (2.9)	0.34
Study eye VA (SD)	0.027 (0.09)	0.057 (0.13)	**0.005**
HORB length estimations			
Foveal B-scans	28.9 (28.2)	26.0 (27.3)	0.31
Non-foveal B-scans	22.2 (26.9)	18.7 (24.2)	0.17
**OCT scan characteristics**	**No pathology**	**Ocular pathology**	***p***
B-scan length (mm)	8.2 (1.8)	10.3 (45.0)	0.61
Eccentricity (mm)	154.8 (360.7)	108.6 (308.9)	0.16
Device type, % (n/N) (Cirrus OCT)	82.1 (96/117)	71.0 (343/483)	**0.02**

Abbreviations: BVS, best vision sphere; VA, visual acuity; logMAR, logarithm of minimal angle resolution; SD, standard deviation.

HORB length

Examples of different HORB values for eyes with and without pathology are shown in **Figure 2**. Note that these percentages should not be interpreted as absolute measures of band length but rather, indicators of outer retinal integrity that can be easily obtained by eye. The mean (SD) foveal HORB length in patients with and without ocular pathology was 26.0% (27.3) and 28.9% (28.2) respectively; for non-foveal B-scans, 18.7% (24.2) and 22.2% (26.9). There was no significant difference in HORB length for foveal and non-foveal B-scans between groups.

Reliability of HORB length estimations

Overall, the reliability of HORB length estimations by the same grader and between graders was good to excellent at foveal and non-foveal scan locations according to pre-defined criteria (ICC=0.75-0.91).[26] A sub-group analysis of reliability by OCT device and B-scan length also found that the intra and inter-rater reliability of HORB estimations for B-scans from Cirrus OCT (ICC=0.77-0.91) and B-scans 5-9mm in length (ICC=0.75-0.91) was good to excellent. For longer B-scans and those captured by Spectralis OCT, the intra and inter-grader reliability was at least moderate (ICC=0.57-0.96) (**Table 2**). Note that the subset of patients with inter-rater reliability data is slightly less than 300 due to processing errors.

For peer review only

Table 2. Intraclass correlation results by device type and B-scan length

**Overall**	**N**		**Intra-rater reliability**	**N**	**Inter-rater reliability***	
					Grader 2	*Grader 3*
	600	*Foveal*	0.81	298	0.79	0.78
		Non-foveal	0.91	296	0.75	0.88
**Device**	**N**		**Intra-rater reliability**	**N**	**Inter-rater reliability**	
Spectralis	151	*Foveal*	0.78	79	0.76	0.80
		Non-foveal	0.91	83	0.67	0.91
Cirrus	379	*Foveal*	0.82	219	0.80	0.78
		Non-foveal	0.91	213	0.77	0.88
**B-scan length**	**N**		**Intra-rater reliability**	**N**	**Inter-rater reliability**	
5-7mm	123	*Foveal*	0.81	94	0.79	0.81
		Non-foveal	0.91	33	0.75	0.90
7-9mm	357	*Foveal*	0.81	177	0.79	0.750.85
		Non-foveal	0.91	227	0.75	
9-11mm	34	*Foveal*	0.57	13	0.80	0.96
		Non-foveal	0.94	21	0.79	0.91
11-15mm†	2	*Foveal*	0.90	2	-	0.83
		Non-foveal	0.88	2	-	0.88
15-17mm	17	*Foveal*	0.76	12	0.67	0.87
		Non-foveal	0.95	13	0.70	0.83

*Inter-rater reliability was calculated from approximately 50% of foveal and non-foveal images from non-identical sub-samples of participants. †Insufficient values to calculate agreement as both graders assigned a value of zero to one of the cases in this group.

Association between HORB length and retinal disease

For foveal B-scans, age (*p<0.001*), VA (*p<0.001*), BVS (*p=0.006*) and a diagnosis of glaucoma (*p=0.03*) were significantly associated with HORB length on univariate analysis. A diagnosis of macular disease or outer retinal disease showed near significant associations (p=0.06, p=0.06 respectively). When all variables which demonstrated a p<0.25 on univariable analysis were included into multivariable analysis, only age, VA and BVS remained significant (**Table 3**).

For non-foveal B-scans, age (*p<0.001*), VA (*p=0.001*), device type (*p=0.01*) and glaucoma diagnosis (*p=0.03*) were significantly associated with HORB length on univariable analysis. A diagnosis of macular disease again showed a near significant association (p=0.06). When all variables demonstrating p<0.25 in univariable analysis were included in multivariable analysis, only age, BVS, eccentricity and device type were significantly associated with HORB length (**Table 3**).

For peer review only - http://bmjopen.bmj.com/site/about/guidelines.xhtml

Together, these results showed no significant association between HORB length and retinal disease. However, age and refractive error significantly affected HORB length at both the foveal and non-foveal locations. VA had a significant effect on foveal HORB length, while B-scan length and eccentricity significantly affected the HORB length of non-foveal B-scans. A sub-analysis of patients without pathology was also conducted to assess for disease-related confounding factors (**Table S3**). Associations were preserved for age for foveal and non-foveal B-scans. For foveal B-scans, associations to visual acuity (p=0.09) and refractive error (p=0.089) were no longer significant, as for eccentricity (p=0.93) and device type (p=0.18) for non-foveal B-scans in eyes without ocular disease, contrasting with the whole cohort analysis.

Table 3. Association between patient/scan characteristics and HORB length

**Univariable analysis**	**Foveal B-scans**		**Non-foveal B-scans**	
	p	*β*†	*p*	*β*
Age (years)	**<0.001**	-0.21	**<0.001**	-0.33
Sex (reference: female)	0.64	-0.02	0.87	0.33
VA (logMAR)	**<0.001**	-0.20	**0.001**	-27.06
BVS (Dioptres)	**0.006**	0.11	0.14	0.51
B-scan length (mm)	0.08	0.07	0.31	-0.04
Eccentricity (mm from fovea)	-	-	0.13	-0.06
Device type‡ (reference: Spectralis OCT)	0.40	0.04	**0.01**	0.11
**Diagnosis** (reference: absence)				
Glaucoma disease	**0.03**	-0.08	0.03	-0.08
Glaucoma suspect	0.81	0.83	0.38	-2.71
Macular disease	0.06	-7.00	0.06	-6.25
Retinal disease	0.40	-3.45	0.61	-1.88
Optic nerve disease	0.83	1.27	0.97	-0.23
Outer retinal disease	0.06	-4.61	0.24	-2.63
**Multiple linear regression analysis§**				
Age (years)	**<0.0001**	-0.22	**<0.0001**	-0.24
VA (logMAR)	**0.001**	-0.13	0.09	-0.07
BVS (Dioptres)	**<0.001**	0.16	**<0.001**	0.12
B-scan length (mm)	0.07	0.07	-	-
Eccentricity (mm from fovea)	-	-	**0.002**	-0.13
Device type (reference: Spectralis OCT)	-	-	**0.002**	0.13
**Diagnosis** (reference: absence)				
Glaucoma disease	0.24	-0.05	0.25	-0.05
Macular disease	0.93	0.005	0.99	-0.001
Outer retinal disease	0.29	-0.06	0.87	-0.01

For peer review only - http://bmjopen.bmj.com/site/about/guidelines.xhtml

BVS = best vision sphere; VA = visual acuity; † Standardised;‡ Spectralis or Cirrus device; § Includes all univariable analysis variables demonstrating $p \leq 0.25$.[27]

DISCUSSION

This study found that HORB length could be assessed reliably from OCT B-scans using magnitude estimation. HORB length estimations were significantly associated with age and visual acuity, however they were not significantly associated with retinal disease after adjusting for covariables. This points to the importance of assessing the utility of outer retinal biomarkers in large, unselected patient populations and controlling for all confounders.

Reliability

HORB length can be reliably assessed using magnitude estimation across different OCT devices and scan protocols in patients with and without retinal pathology. Our results show that the reliability of estimating HORB length at the fovea was good to excellent across two major commercially available OCT devices and scan lengths of 9mm or less for an unselected population exhibiting a range of ocular pathology. Moreover, intra- and inter-rater reliability of HORB estimations was at least moderate for all devices or scan lengths tested at both foveal and non-foveal B-scan locations. At non-foveal locations, intra-rater reliability of magnitude estimations was excellent for both OCT devices and all scan lengths and but only moderate to good between graders, with the exception of B-scans captured by Spectralis OCT and B-scan lengths of 7mm or less. The greater axial resolution of Spectralis OCT B-scans (versus Cirrus OCT[23]) and increased difficulty of estimating HORB length across longer B-scan likely accounts for these findings.

Our results are in line with the few previous reports that found ELM and EZ integrity measurements reliable.[7, 28] Indeed, other applications of magnitude estimation in eye care such as optic nerve cup-to-disc ratio estimation [20] and bulbar conjunctival redness[21] grading, also show good reliability and are routinely used in clinical practice. Whilst categorical approaches have been used previously to evaluate outer retinal integrity, many do not report reliability and the continuous nature of magnitude estimations may be advantageous over these methods as visual assessment of clinical features using scales with smaller increments has been shown to improve inter and intra-rater reliability.[29, 30]

Association between outer retinal disruptions and clinical outcomes

We found visual acuity was significantly associated with foveal HORB length in an unselected patient population. This supports previous suggestions that outer retinal integrity could be a useful surrogate biomarker of visual acuity,[31] particularly in severe retinal disease such as retinitis pigmentosa where visual acuity measures become less repeatable or unobtainable.[32] HORB length was also significantly associated with age in both foveal and non-foveal scans, consistent with other reports,[10, 12] emphasising the need for age-correction when assessing image-based biomarkers.

We also identified refractive error, eccentricity and device type as additional covariables affecting HORB length that have not been previously reported. However, in our subset analysis of eyes without ocular disease, only age remained significantly associated with both foveal and non-foveal HORB

For peer review only - http://bmjopen.bmj.com/site/about/guidelines.xhtml

length while refractive error significantly affected non-foveal HORB but not foveal HORB length. These differences could be explained by the effects of cataracts and pseudophakia on visual acuity and refraction, particularly as the diseased group was statistically older. The lack of association between B-scan eccentricity and device type to HORB length in eyes without ocular pathology likely stems from differences in clinical decision-making processes between groups as non-foveal B-scans are selected randomly in eyes without pathology and OCT devices may be chosen based on imaging capabilities. For example, peripheral OCT B-scans are more easily obtained using Spectralis OCT devices[33] and thus are more likely to be used when peripheral pathology is identified.

Differences in cone density with axial length[34] may explain the association between refractive error and HORB length as band reflectivity varies with cone density[35] and affects band visibility. Lower cone densities are also found at greater eccentricities which may explain the negative correlation between HORB length and eccentricity in non-foveal scans. This effect was only significant for non-foveal scans, perhaps as foveal scans were arbitrarily selected and did not necessarily include outer retinal disruptions. Considering the number of studies emphasizing the prognostic potential of outer retinal disruptions[7, 8] and relationships to other clinical outcomes, the significant covariables identified in this study will be highly relevant to future validation of these biomarkers in retinal disease.

Other groups have reported significant reduction of ellipsoid zone intensity and interdigitation zone area in glaucomatous eyes compared to age-matched normal eyes.[36, 37] We also found that glaucoma disease was significantly associated with HORB length but this was not maintained when we adjusted for covariables in multivariate analysis. Similarly, outer retinal disease such as subretinal fluid[11] and early AMD[4] have been associated with compromised HORB integrity, yet we did not find a significant association after adjusting for covariables. The disparity between our study findings and previous evidence may stem from the recruitment of study participants from a non-tertiary care centre rather than hospital or ophthalmology clinic settings[2, 5, 7, 11] where retinal disease patients are typically at more advanced stages and exhibit a higher degree of outer retinal disruption.[3, 5] Thus, examining magnitude estimation of HORB length in a population with more severe retinal disease may be useful to elucidate the strength of its association to disease.

Mean HORB length estimations were also lower than expected, particularly in the group without ocular pathology where we expected to see intact HORB at least throughout the foveal scan. The strong inverse correlation between age, visual acuity, B-scan length and eccentricity and HORB length suggests that these factors likely contributed to this result as the effect was sustained after adjusting for disease status. These findings again, emphasise the importance of adjusting for covariables when measuring outer retinal band biomarkers.

Strengths and limitations

A key strength to the study was using a consecutive recruitment strategy to identify an unselected patient population, which was more representative of individuals attending primary eye care practices. This therefore improves the generalisability of findings by enabling a true estimation of associations between HORB length, clinical outcomes and covariables. However, this also created limitations around sub-group sample sizes due to the low prevalence of retinal disease in patients referred to CFEH.[22] As such, further work is needed to evaluate outer retinal disruptions in specific disease types and may benefit from oversampling target sub-groups.

For peer review only - http://bmjopen.bmj.com/site/about/guidelines.xhtml

Compared to previous groups, we used a reliable psychophysical technique to assess outer retinal integrity by eye which uses a continuous rather than categorical scale (e.g. absent/discontinuous versus present/continuous)[38, 39] This is advantageous as visual assessment of clinical features using scales with smaller increments has been shown to improve inter and intra-rater reliability.[29, 30] A limitation of the study was that the ELM was not included as part of the HORB assessment even though ELM disruptions have also been correlated with retinal disease.[3, 13, 14] This structure was not included in the grading task as the intensity of the ELM is markedly reduced compared to the other bands and may be difficult to reliably detect by visual estimation. Given our findings, it may be worthwhile evaluating the impact of patient and imaging factors on ELM integrity in future investigations.

Another limitation was that all estimations were performed manually and no artificial (AI) intelligence-driven methods of quantifying HORB length were explored. While AI techniques have shown increasing promise for clinical applications,[40] the implementation of AI-driven tools in primary or tertiary eye care clinics is presently low with only 15.7% of ophthalmologists currently using AI-derived algorithms in daily practice.[41] Primary eye care practices are also known to have slow uptake of new clinical tools[42] with clinicians still reporting reservations about AI decision-support technology.[43] Thus, magnitude estimation was pursued as a highly accessible technique that requires no specialised equipment to initially assess whether subjective outer retinal integrity gradings are indeed associated with retinal disease, as described by other groups, and whether the method can be used to establish a reliable ground truth. Our results suggest that the clinical relevance of outer retinal integrity assessments to retinal disease requires further investigation using a larger pool of raters and greater representation of disease subgroups in which associations neared significance. If significant associations to disease are confirmed, then magnitude estimations should be validated against computationally derived measurements of outer retinal band length before potential deployment to clinical settings.

CONCLUSION

We show that outer retinal band length can be reliably assessed using magnitude estimation and may be useful as a surrogate biomarker of visual acuity. Importantly, this study is the first to assess the reliability of measuring HORB integrity beyond the fovea and demonstrates that HORB length at non-foveal locations can be measured reliably using magnitude estimation. Several factors including age, refractive error, eccentricity and device type affected HORB length estimations and highlights the importance of adjusting for covariables when evaluating HORB disruptions in retinal disease to ensure true associations are obtained.

*** ***

COMPETING INTERESTS

The authors declare no competing interests. This work was presented as a conference abstract at the XXV Biennial Meeting of the International Society for Eye Research, February 19-23, 2023, Gold Coast, Queensland, Australia.

For peer review only - http://bmjopen.bmj.com/site/about/guidelines.xhtml

FUNDING

This work was supported by funding from the National Health and Medical Research Council (NHMRC #1174385, #1186915) and the Research Training Program scholarship. Guide Dogs NSW/ACT provides support for the Centre for Eye Health (the clinic from which patient data was obtained).

CONTRIBUTORS

RC, LNS, AL and MK were involved in study conception and design. HW and RC performed data collection and RC analyzed and drafted the manuscript. RC, LNS, AL and MK participated in the interpretation of results and revision of the manuscript. The corresponding author confirms that all listed authors meet authorship criteria and no other meeting criteria have been omitted.

ACKNOWLEDGEMENTS

The authors thank Natalie Eshow, Meri Galoyan, Helene Ly and Matt Trinh for their assistance on data collection and statistical analysis.

DATA AVAILABILITY STATEMENT

Data described in the manuscript and supplementary material will not be made publicly available. Further information about procedures for obtaining and accessing data from Centre for Eye Health is described at https://www.centreforeyehealth.com.au/research/ (email: enquiries@cfeh.com.au).

REFERENCES

1. Cetin EN, Parca O, Akkaya HS, *et al.* Association of retinal biomarkers and choroidal vascularity index on optical coherence tomography using binarization method in retinitis pigmentosa. Graefe's Archive for Clinical Experimental Ophthalmology 2020;**258**(1):23-30
2. Ojima Y, Tsujikawa A, Yamashiro K, *et al.* Restoration of outer segments of foveal photoreceptors after resolution of central serous chorioretinopathy. Jpn J Ophthalmol 2010;**54**(1):55-60 doi: 10.1007/s10384-009-0766-4[published Online First: Epub Date]|.
3. Jain A, Saxena S, Khanna VK, *et al.* Status of serum VEGF and ICAM-1 and its association with external limiting membrane and inner segment-outer segment junction disruption in type 2 diabetes mellitus. Molecular vision 2013;**19**:1760
4. Wu Z, Ayton LN, Guymer RH, *et al.* Relationship Between the Second Reflective Band on Optical Coherence Tomography and Multifocal Electroretinography in Age-Related Macular Degeneration. Investigative Ophthalmology & Visual Science 2013;**54**(4):2800-06 doi: 10.1167/iovs.13-11613[published Online First: Epub Date]|.
5. Schumann RG, Compera D, Schaumberger MM, *et al.* Epiretinal membrane characteristics correlate with photoreceptor layer defects in lamellar macular holes and macular

For peer review only - http://bmjopen.bmj.com/site/about/guidelines.xhtml

pseudoholes. Retina 2015;**35**(4):727-35 doi: 10.1097/IAE.0000000000000375[published Online First: Epub Date]|.

6. Das R, Spence G, Hogg RE*, et al.* Disorganization of Inner Retina and Outer Retinal Morphology in Diabetic Macular Edema. JAMA Ophthalmol 2018;**136**(2):202-08 doi: 10.1001/jamaophthalmol.2017.6256[published Online First: Epub Date]|.
7. Oishi A, Shimozono M, Mandai M*, et al.* Recovery of photoreceptor outer segments after anti-VEGF therapy for age-related macular degeneration. Graefe's archive for clinical and experimental ophthalmology 2013;**251**(2):435-40
8. Muftuoglu IK, Mendoza N, Gaber R*, et al.* Integrity of outer retinal layers after resolution of central involved diabetic macular edema. Retina (Philadelphia, Pa.) 2017;**37**(11):2015
9. Sundaram V, Wilde C, Aboshiha J*, et al.* Retinal structure and function in achromatopsia: implications for gene therapy. Ophthalmology 2014;**121**(1):234-45 doi: 10.1016/j.ophtha.2013.08.017[published Online First: Epub Date]|.
10. Gin TJ, Wu Z, Chew SKH*, et al.* Quantitative Analysis of the Ellipsoid Zone Intensity in Phenotypic Variations of Intermediate Age-Related Macular Degeneration. Investigative Ophthalmology & Visual Science 2017;**58**(4):2079-86 doi: 10.1167/iovs.16-20105[published Online First: Epub Date]|.
11. Hood DC, Zhang X, Ramachandran R*, et al.* The inner segment/outer segment border seen on optical coherence tomography is less intense in patients with diminished cone function. Investigative ophthalmology & visual science 2011;**52**(13):9703-09
12. Arepalli S, Traboulsi EI, Ehlers JP. Ellipsoid zone mapping and outer retinal assessment in Stargardt disease. Retina 2018;**38**(7):1427
13. Nakamura T, Ueda-Consolvo T, Oiwake T*, et al.* Correlation between outer retinal layer thickness and cone density in patients with resolved central serous chorioretinopathy. Graefe's Archive for Clinical and Experimental Ophthalmology 2016;**254**(12):2347-54
14. Wu Z, Ayton LN, Guymer RH*, et al.* Second reflective band intensity in age-related macular degeneration. Ophthalmology 2013;**120**(6):1307-08. e1
15. Gescheider GA. Psychophysical scaling. Annual review of psychology 1988;**39**(1):169-200
16. Verrillo RT. Stability of line-length estimates using the method of absolute magnitude estimation. Perception Psychophysics 1983;**33**(3):261-65
17. Gescheider GA, Hughson BA. Stimulus context and absolute magnitude estimation: A study of individual differences. Perception & Psychophysics 1991;**50**(1):45-57
18. Stevens SS, Marks LE. *Psychophysics: Introduction to its perceptual, neural, and social prospects*: Routledge, 2017.
19. Stevens SS. On the psychophysical law. Psychol Rev 1957;**64**(3):153-81 doi: 10.1037/h0046162[published Online First: Epub Date]|.
20. Feuer WJ, Parrish II RK, Schiffman JC*, et al.* The Ocular Hypertension Treatment Study: reproducibility of cup/disk ratio measurements over time at an optic disc reading center. American journal of ophthalmology 2002;**133**(1):19-28
21. Chong T, Simpson T, Fonn D. The repeatability of discrete and continuous anterior segment grading scales. Optometry and Vision Science 2000;**77**(5):244-51
22. Wang H, Kalloniatis M. Clinical outcomes of the Centre for Eye Health: an intra-professional optometry-led collaborative eye care clinic in Australia. Clinical Experimental Optometry 2021:1-10
23. Leite MT RH, Zangwill LM, Weinreb RN, Medeiros FA. Comparison of the diagnostic accuracies of the Spectralis, Cirrus, and RTVue optical coherence tomography devices in glaucoma. Ophthalmology 2011;**118**(7):1334-9
24. Age-Related Eye Disease Study Research Group. A simplified severity scale for age-related macular degeneration: AREDS report no. 18. Archives of ophthalmology 2005;**123**(11):1570

For peer review only

For peer review only - http://bmjopen.bmj.com/site/about/guidelines.xhtml

25. Staurenghi G, Sadda S, Chakravarthy U*, et al.* Proposed lexicon for anatomic landmarks in normal posterior segment spectral-domain optical coherence tomography: the IN• OCT consensus. Ophthalmology 2014;**121**(8):1572-78
26. Koo TK, Li MY. A Guideline of Selecting and Reporting Intraclass Correlation Coefficients for Reliability Research. J Chiropr Med 2016;**15**(2):155-63 doi: 10.1016/j.jcm.2016.02.012[published Online First: Epub Date]|.
27. Bursac Z, Gauss CH, Williams DK*, et al.* Purposeful selection of variables in logistic regression. Source code for biology and medicine 2008;**3**(1):1-8
28. Reibaldi M, Parravano M, Varano M*, et al.* Foveal microstructure and functional parameters in lamellar macular hole. American journal of ophthalmology 2012;**154**(6):974-80. e1
29. Fowler PV, Al-Ani AH, Thompson JM. Comparison of reliability of categorical and continuous scales for radiographic assessments of bone infill following secondary alveolar bone grafting. The Cleft Palate-Craniofacial Journal 2018;**55**(2):269-75
30. Masmali AM, Murphy PJ, Purslow C. Development of a new grading scale for tear ferning. Contact Lens and Anterior Eye 2014;**37**(3):178-84
31. Sousa K, Fernandes T, Gentil R*, et al.* Outer retinal layers as predictors of visual acuity in retinitis pigmentosa: a cross-sectional study. Graefe's Archive for Clinical and Experimental Ophthalmology 2019;**257**:265-71
32. Kiser AK, Mladenovich D, Eshraghi F*, et al.* Reliability and consistency of visual acuity and contrast sensitivity measures in advanced eye disease. Optometry and vision science 2005;**82**(11):946-54
33. Aumann S, Donner S, Fischer J*, et al.* Optical coherence tomography (OCT): principle and technical realization. High resolution imaging in microscopy and ophthalmology: new frontiers in biomedical optics 2019:59-85
34. Chui TYP, Song H, Burns SA. Individual variations in human cone photoreceptor packing density: variations with refractive error. Investigative ophthalmology & visual science 2008;**49**(10):4679-87
35. Flores M, Debellemanière G, Bully A*, et al.* Reflectivity of the outer retina on spectral-domain optical coherence tomography as a predictor of photoreceptor cone density. American Journal of Ophthalmology 2015;**160**(3):588-95. e2
36. Ha A, Kim YK, Jeoung JW*, et al.* Ellipsoid zone change according to glaucoma stage advancement. American journal of ophthalmology 2018;**192**:1-9
37. Ha A, Kim YK, Lee J*, et al.* Interdigitation Zone Change According to Glaucoma-Stage Advancement. Invest Ophthalmol Vis Sci 2020;**61**(4):20 doi: 10.1167/iovs.61.4.20[published Online First: Epub Date]|.
38. Akagi-Kurashige Y, Tsujikawa A, Oishi A*, et al.* Relationship between retinal morphological findings and visual function in age-related macular degeneration. Graefe's archive for clinical and experimental ophthalmology 2012;**250**(8):1129-36
39. Landa G, Su E, Garcia PM*, et al.* Inner segment–outer segment junctional layer integrity and corresponding retinal sensitivity in dry and wet forms of age-related macular degeneration. Retina 2011;**31**(2):364-70
40. Keenan TD, Clemons TE, Domalpally A*, et al.* Retinal Specialist versus Artificial Intelligence Detection of Retinal Fluid from OCT: Age-Related Eye Disease Study 2: 10-Year Follow-On Study. Ophthalmology 2021;**128**(1):100-09
41. Scheetz J, Koklanis K, McGuinness M*, et al.* Gaze behaviour and accuracy among novice and glaucoma specialist orthoptists during optic disc examination: A cross sectional study. Australian Orthoptic Journal 2019;**51**:17-24
42. Cheung R, Ho S, Ly A. Optometrists' attitudes toward using OCT angiography lag behind other retinal imaging types. Ophthalmic and Physiological Optics 2023
43. Safi S, Thiessen T, Schmailzl KJ. Acceptance and resistance of new digital technologies in medicine: qualitative study. JMIR research protocols 2018;**7**(12):e11072

For peer review only - http://bmjopen.bmj.com/site/about/guidelines.xhtml

FIGURE LEGENDS

Figure 1. *(A)* High definition, Cirrus B-scan of a healthy fovea with *(B)* the hyperreflective outer retinal bands (HORB) assessed for outer retinal integrity: ellipsoid zone (red), interdigitation zone (blue) and RPE/ Bruch's membrane complex (green). The external limiting membrane (white arrows, panel B) was not included in our definition of HORB.

Figure 2. Examples of foveal (I) and worst B-scans without (II) and with visual representation of outer retinal band estimations performed by eye (III; ellipsoid zone (red), interdigitation zone (blue) and RPE/ Bruch's membrane complex (green) for eyes with *(row A)* no retinal pathology (100%), *(row B)* central serous chorioretinopathy (0%) and *(row C)* multifocal pattern dystrophy (10%). Estimation of HORB length is indicated in the top right-hand corner of each panel.

For peer review only

For peer review only - http://bmjopen.bmj.com/site/about/guidelines.xhtml

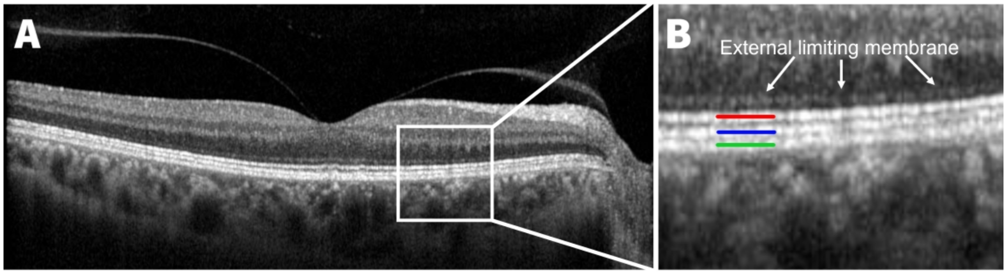

1309x363mm (72 x 72 DPI)

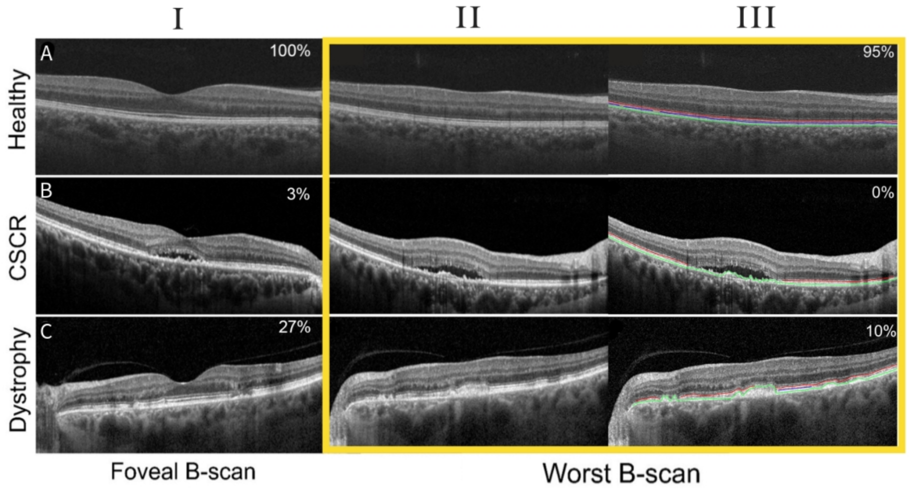

709x466mm (72 x 72 DPI)

Supplementary Material

Methods – OCT B-scan grading instructions

OCT B-scans were graded using the following instructions: '*Not counting the area directly under the optic nerve (if your image contains an optic nerve), what percentage of the horizontal length of the scan shows three complete and continuous hyperreflective lines in the retinal layers between the myoid zone and choriocapillaris? Ignore padding between around the OCT B-scan, shadowing and areas under the optic nerve.*' All images were reviewed in full-screen mode at 100% magnification, maximal brightness and standard contrast settings (Asus Vivobook S15).

Table S1: Diagnosis classification

Diagnosis category	Criteria for inclusion
Glaucoma disease	Glaucoma diagnosis recorded in clinical record
Glaucoma suspect	• Glaucoma risk requiring further work-up due to historical risk factors and/or detection of structural and/or functional signs suggestive of glaucoma.<br>• Narrow angles requiring further monitoring
Optic nerve disease	Non-glaucomatous optic nerve pathology
Macular disease	• AMD<br>• Pachychoroid spectrum disease<br>• Other macular pathology e.g. retinal dystrophy, epiretinal membranes<br>• Non-specific macular pathology e.g. isolated pigmented disruption or drusen
Retinal disease	• Diabetic retinopathy<br>• Myopia-related complications<br>• Extra-macular retinal pathology unrelated to myopia or diabetes
Other	• Corneal degenerations<br>• Visual symptoms or headaches unexplained by clinical findings<br>• Pigmented lesion<br>• Anterior eye disease including dry eye, conjunctival lesions or other pathology unrelated to the cornea<br>• Vitreous anomalies
Multiple diagnoses	Diagnosed with multiple conditions in different categories
No ocular pathology	No ocular pathology noted in clinician report including druplets, cataracts, refractive errors and physiological cupping or nerve crowding.
Outer retinal disease	Diagnosis of macular and/or retinal disease

For peer review only - http://bmjopen.bmj.com/site/about/guidelines.xhtml

Table S2: Patient diagnoses

**Diagnostic category**	(%, n/N)	Foveal HORB length (mean, SD)	Non-foveal HORB length (mean, SD)
Glaucoma suspect	23.8 (143/600)	27.9 (27.4)	18.2 (24.8)
Macular disease	16.5 (99/600)	21.9 (26.4)	16.0 (21.8)
Retinal disease	12.0 (72/600)	25.5 (29.4)	20.3 (26.7)
Multiple diagnoses	10.8 (65/600)	21.8 (24.6)	16.8 (20.3)
Other diagnoses	9.3 (56/600)	31.1 (29.7)	23.5 (28.0)
Optic nerve disease	4.5 (27/600)	30.2 (25.3)	22.0 (21.4)
Glaucoma disease	3.5 (21/600)	14.9 (19.1)	9.4 (19.5)
No ocular pathology	19.5 (117/600)	28.9 (28.2)	22.2 (26.9)

Figure S1: pie chart of OCT scan patterns from which B-scans were extracted. *Other scan types include B-scans from Spectralis 20x20 scan patterns and single line B-scans.

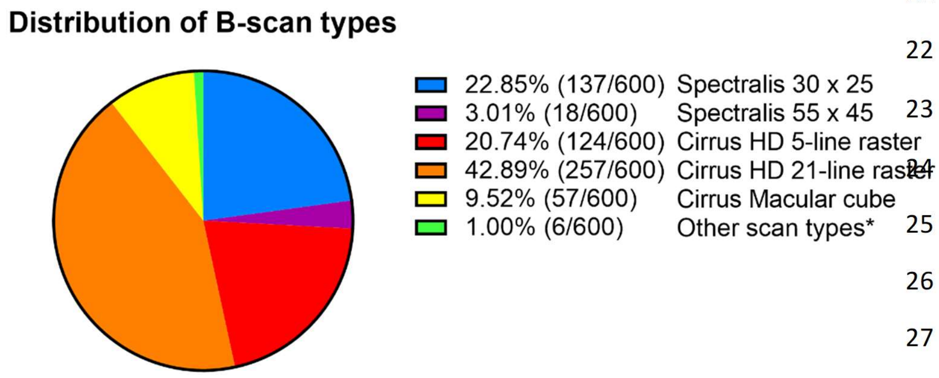

Table S3: Association between patient/scan characteristics and HORB length for patients without ocular pathology

**Univariable analysis**	**Foveal B-scans**		**Non-foveal B-scans**	
	p	*β*	*p*	*β*
Age (years)	**0.03**	-0.20	**<0.0001**	-0.22
Sex (reference: female)	0.80	-0.02	0.41	-0.08
VA (logMAR)	0.09	-0.16	0.06	-0.08
BVS (Dioptres)	0.89	0.01	**0.01**	0.12
B-scan length (mm)	0.69	-0.04	0.28	-0.04
Eccentricity (mm from fovea)	-	-	0.93	0.01
Device type‡ (reference: Spectralis OCT)	0.84	-0.02	0.13	0.14
**Diagnosis** (reference: absence)				
Glaucoma disease	-	-	-	-
Glaucoma suspect	-	-	-	-
Macular disease	-	-	-	-
Retinal disease	-	-	-	-

For peer review only - http://bmjopen.bmj.com/site/about/guidelines.xhtml

Optic nerve disease	-	-	-	-
Outer retinal disease	-	-	-	-
**Multiple linear regression analysis**				
Age (years)	**0.03**	-0.16	**0.05**	-0.20
VA (logMAR)	0.09	-0.16	0.38	-0.09
BVS (Dioptres)	-	-	**0.04**	0.21
B-scan length (mm*)*	-	-	-	-
Eccentricity (mm from fovea)	-	-	-	-
Device type (reference: Spectralis OCT)	-	-	0.18	0.13
**Diagnosis** (reference: absence)	-	-	-	-
Glaucoma disease				
Macular disease	-	-	-	-
Outer retinal disease	-	-	-	-

For peer review only

STROBE Statement—Checklist of items that should be included in reports of ***cross-sectional studies***

	Item No	Recommendation	Page No
**Title and abstract**	1	(*a*) Indicate the study's design with a commonly used term in the title or the abstract	2
		(*b*) Provide in the abstract an informative and balanced summary of what was done and what was found	2
**Introduction**			
Background/rationale	2	Explain the scientific background and rationale for the investigation being reported	3
Objectives	3	State specific objectives, including any prespecified hypotheses	3
**Methods**			
Study design	4	Present key elements of study design early in the paper	3
Setting	5	Describe the setting, locations, and relevant dates, including periods of recruitment, exposure, follow-up, and data collection	3
Participants	6	(*a*) Give the eligibility criteria, and the sources and methods of selection of participants	3-4
Variables	7	Clearly define all outcomes, exposures, predictors, potential confounders, and effect modifiers. Give diagnostic criteria, if applicable	4-5
Data sources/ measurement	8*	For each variable of interest, give sources of data and details of methods of assessment (measurement). Describe comparability of assessment methods if there is more than one group	4-5
Bias	9	Describe any efforts to address potential sources of bias	3
Study size	10	Explain how the study size was arrived at	5
Quantitative variables	11	Explain how quantitative variables were handled in the analyses. If applicable, describe which groupings were chosen and why	5
Statistical methods	12	(*a*) Describe all statistical methods, including those used to control for confounding	5
		(*b*) Describe any methods used to examine subgroups and interactions	5
		(*c*) Explain how missing data were addressed	3-4
		(*d*) If applicable, describe analytical methods taking account of sampling strategy	
		(*e*) Describe any sensitivity analyses	4
**Results**			
Participants	13*	(a) Report numbers of individuals at each stage of study—eg numbers potentially eligible, examined for eligibility, confirmed eligible, included in the study, completing follow-up, and analysed	3 & 5
		(b) Give reasons for non-participation at each stage	4-4
		(c) Consider use of a flow diagram	N/A
Descriptive data	14*	(a) Give characteristics of study participants (eg demographic, clinical, social) and information on exposures and potential confounders	5-6
		(b) Indicate number of participants with missing data for each variable of interest	N/A
Outcome data	15*	Report numbers of outcome events or summary measures	6-7
6Main results	16	(*a*) Give unadjusted estimates and, if applicable, confounder-adjusted estimates and their precision (eg, 95% confidence interval). Make clear which confounders were adjusted for and why they were included	7-8

For peer review only - http://bmjopen.bmj.com/site/about/guidelines.xhtml

		(*b*) Report category boundaries when continuous variables were categorized	6
		(*c*) If relevant, consider translating estimates of relative risk into absolute risk for a meaningful time period	N/A
Other analyses	17	Report other analyses done—eg analyses of subgroups and interactions, and sensitivity analyses	6-7
**Discussion**			
Key results	18	Summarise key results with reference to study objectives	8
Limitations	19	Discuss limitations of the study, taking into account sources of potential bias or imprecision. Discuss both direction and magnitude of any potential bias	9
Interpretation	20	Give a cautious overall interpretation of results considering objectives, limitations, multiplicity of analyses, results from similar studies, and other relevant evidence	8-9
Generalisability	21	Discuss the generalisability (external validity) of the study results	8
**Other information**			
Funding	22	Give the source of funding and the role of the funders for the present study and, if applicable, for the original study on which the present article is based	10

*Give information separately for exposed and unexposed groups.

Note: An Explanation and Elaboration article discusses each checklist item and gives methodological background and published examples of transparent reporting. The STROBE checklist is best used in conjunction with this article (freely available on the Web sites of PLoS Medicine at http://www.plosmedicine.org/, Annals of Internal Medicine at http://www.annals.org/, and Epidemiology at http://www.epidem.com/). Information on the STROBE Initiative is available at www.strobe-statement.org.

For peer review only

For peer review only - http://bmjopen.bmj.com/site/about/guidelines.xhtml
